# Correction: Prevalence, infection intensity, and risk factors of soil-transmitted helminthiasis and intestinal schistosomiasis among schoolchildren in Southern Ethiopia

**DOI:** 10.1371/journal.pntd.0014682

**Published:** 2026-08-28

**Authors:** The PLOS Neglected Tropical Diseases Staff

In the Data Collection Tools and Procedures section, the subheading "Schistosoma mansoni” is incorrect. The correct subheading is: Parasitological Examination.

The publisher apologizes for the error.
