# Supplementary material for: Prevalence, infection intensity, and risk factors of soil-transmitted helminthiasis and intestinal schistosomiasis among schoolchildren in Southern Ethiopia
Source: PLoS Negl Trop Dis. 2026 Mar 18;20(3):e0014115. doi: 10.1371/journal.pntd.0014115 (PMC13016474; doi:10.1371/journal.pntd.0014115)
Supplement: S2 Table — (DOCX) [file pntd.0014115.s002.docx]

| School Name  ______________________ | Child Name  _________________________ | ID  _______________________ |
| --- | --- | --- |
| Grade  ______________________ | Interviewer Name  _________________________ | Date of Interview (dd/mm/yyyy)  ________________________ |

| **Part one questioner on Socio-demographic part** | | | **Response** | **Code** |
| --- | --- | --- | --- | --- |
| Age | | _______years |  | A1 |
| Sex | | Male  Female | 1  2 | A2 |
| Religion | | Orthodox  Muslim  Protestant  Catholic  Other (specify),_________________________ | 1  2  3  4 | A3 |
| Residence | | Urban  Semi Urban  Rural | 1  2  3 | A4 |
| Educational status of mother | | No formal education  Primary school  Secondary school  College and above | 1  2  3  4 | A6 |
| Educational status of father | | No formal education  Primary  Secondary  College and above | 1  2  3  4 | A7 |
| What is the highest level of family education that have completed? | | Primary (1-8)  Secondary (9-12)  Diploma and above | 1  2  3 | A9 |
| The occupation status of your mother? | | Housewife  Government Employee  Private Business  Students  Others specify_________ | 1  2  3  4  5 | A10 |
| The occupation status of your father? | | Government Employee  Daily laborer  Private business  Students  Others specify_________ | 1  2  3  4  5 | A11 |
| Living condition of child | | Living with parents/partners  Living with relative  Living with friends in a rental house  Living alone in a rented house  Others(Specify)_______ | 1  2  3  4  5 | A12 |
| Family size including you? | | _______ |  | A13 |
| How many sopuses /wives have the house head | | single wife  polygamy | 1  2 | A14 |
| **Part two questioner on Wealth index** | | | **Response** | **code** |
| Does your household have the following properties? | Functioning radio/Tape recorder/CD player | Yes  No | 1  0 | A16 |
|  | Functioning television | Yes  No | 1  0 | A17 |
|  | Gas Stove | Yes  No | 1  0 | A18 |
|  | Electric stove | Yes  No | 1  0 | A19 |
|  | Motor Cycle | Yes  No | 1  0 | A20 |
|  | Cart/ Gari | Yes  No | 1  0 | A21 |
|  | Watch (Hand/Wall) | Yes  No | 1  0 | A22 |
|  | Mobile phone | Yes  No | 1  0 | A23 |
|  | Bicycle | Yes  No | 1  0 | A24 |
|  | Sofa | Yes  No | 1  0 | A25 |
|  | Sponge/foam mattress | Yes  No | 1  0 | A26 |
|  | Chair/Stool | Yes  No | 1  0 | A27 |
|  | Generator/ Solar | Yes  No | 1  0 | A28 |
|  | Milling | Yes  No | 1  0 | A29 |
|  | Car | Yes  No | 1  0 | A30 |
|  | Refrigerator | Yes  No | 1  0 | A31 |
|  | Bajaj | Yes  No | 1  0 | A32 |
|  | Own house | Yes  No | 1  0 | A33 |

**Food frequency questioner**

**Instruction-**Dear respondent please take few moment to memorize the food and drinks you ate within the last three month .These relate to your daily use of food items and also food consumed out of home, e.g in aresturant, with friends outside home and at scho-ol. I will say the food if you consumed the food type you will tell me how often you ate over the last three month.

| Thinking back on the last three months, please tell me how often you consumed each of the following items. You can tell me in terms of times per day, per week or per month. **ENTER 0 IF NEVER CONSUMED THE ITEM(cereals and legumes** | | | | | |
| --- | --- | --- | --- | --- | --- |
| K1. Teff | 1. Never or<1* per month 2. 1 * per month 3. 2-3 * per month 4. 1 * per week 5. 2-3 * per week 6. 4-6 * per week 7. Every day 8. More onetime per day |  | K2. Maize | 1. Never or<1*permonth 2. 1 * per month 3. 2-3 * per month 4. 1 * per week 5. 2-3 * per week 6. 4-6 * per week 7. Every day 8. More onetime per day |  |
| K3. Barley | 1. Never or<1* per month 2. 1 * per month 3. 2-3 * per month 4. 1 * per week 5. 2-3 * per week 6. 4-6 * per week 7. Every day 8. More onetime per day |  | K4. Wheat,  including bread | 1. Never or<1* per month 2. 1 * per month 3. 2-3 * per month 4. 1 * per week 5. 2-3 * per week 6. 4-6 * per week 7. Every day 8. More onetime per day |  |
| K5.Sorghum/millet | 1. Never or<1* per month 2. 1 * per month 3. 2-3 * per month 4. 1 * per week 5. 2-3 * per week 6. 4-6 * per week 7. Every day 8. More onetime per day |  | K6. Rice | 1. Never or<1* per month 2. 1 * per month 3. 2-3 * per month 4. 1 * per week 5. 2-3 * per week 6. 4-6 * per week 7. Every day 8. More onetime per day |  |

| Thinking back on the last three months, please tell me how often you consumed each of the following items. You can tell me in terms of times per day, per week or per month. **ENTER 0 IF NEVER CONSUMED THE ITEM** | | | | | |
| --- | --- | --- | --- | --- | --- |
| K7.Pasta(Macroni) | 1. Never or<1* per month 2. 1 * per month 3. 2-3 * per month 4. 1 * per week 5. 2-3 * per week 6. 4-6 * per week 7. Every day   8.More onetime per day |  | K8. Ye/aja kinche(oats) | 1. Never or<1* per month 2. 1 * per month 3. 2-3 * per month 4. 1 * per week 5. 2-3 * per week 6. 4-6 * per week 7. Every day   8.More onetime per day |  |
| K9. Lentil | 1. Never or<1* per month 2. 1 * per month 3. 2-3 * per month 4. 1 * per week 5. 2-3 * per week 6. 4-6 * per week 7. Every day 8. More onetime per day |  | K10.Barely(poriadge,Genfo) | 1. Never or<1* per month 2. 1 * per month 3. 2-3 * per month 4. 1 * per week 5. 2-3 * per week 6. 4-6 * per week 7. Every day 8. More onetime per day |  |
| K11.Chick peas(shinbera) | 1. Never or<1* per month 2. 1 * per month 3. 2-3 * per month 4. 1 * per week 5. 2-3 * per week 6. 4-6 * per week 7. Every day 8. More onetime per day |  | K12.Beans | 1. Never or<1* per month 2. 1 * per month 3. 2-3 * per month 4. 1 * per week 5. 2-3 * per week 6. 4-6 * per week 7. Every day 8. More onetime per day |  |

| Thinking back on the last three months, please tell me how often you consumed each of the following items. You can tell me in terms of times per day, per week or per month. **ENTER 0 IF NEVER CONSUMED THE ITEM(Roots and tubers)** | | | | | |
| --- | --- | --- | --- | --- | --- |
| K13.Kocho | 1. Never or<1* per month 2. 1 * per month 3. 2-3 * per month 4. 1 * per week 5. 2-3 * per week 6. 4-6 * per week 7. Every day   8.More onetime per day |  | K14. Sweet potatoes | 1. Never or<1* per month 2. 1 * per month 3. 2-3 * per month 4. 1 * per week 5. 2-3 * per week 6. 4-6 * per week 7. Every day   8.More onetime per day |  |
| K15. Carrot | 1. Never or<1* per month 2. 1 * per month 3. 2-3 * per month 4. 1 * per week 5. 2-3 * per week 6. 4-6 * per week 7. Every day 8. More onetime per day |  | K16.Potatoe | 1. Never or<1* per month 2. 1 * per month 3. 2-3 * per month 4. 1 * per week 5. 2-3 * per week 6. 4-6 * per week 7. Every day 8. More onetime per day |  |

| Thinking back on the last three months, please tell me how often you consumed each of the following items. You can tell me in terms of times per day, per week or per month. **ENTER 1 IF NEVER CONSUMED THE ITEM(vegetables)** | | | | | |
| --- | --- | --- | --- | --- | --- |
| K17.Chard (kosta) | 1. Never or<1* per month 2. 1 * per month 3. 2-3 * per month 4. 1 * per week 5. 2-3 * per week 6. 4-6 * per week 7. Every day 8. More onetime per day |  | K18.Cabbage | 1. Never or<1* per month 2. 1 * per month 3. 2-3 * per month 4. 1 * per week 5. 2-3 * per week 6. 4-6 * per week 7. Every day 8. More onetime per day |  |
| K19.Kale(tikur gomen) | 1. Never or<1* per month 2. 1 * per month 3. 2-3 * per month 4. 1 * per week 5. 2-3 * per week 6. 4-6 * per week 7. Every day 8. More onetime per day |  | K20.Tomatoe | 1. Never or<1* per month 2. 1 * per month 3. 2-3 * per month 4. 1 * per week 5. 2-3 * per week 6. 4-6 * per week 7. Every day 8. More onetime per day |  |
| K21.Pumkin(duba) | 1. Never or<1* per month 2. 1 * per month 3. 2-3 * per month 4. 1 * per week 5. 2-3 * per week 6. 4-6 * per week 7. Every day 8. More onetime per day |  | K22. Green beans (fosoliya) | 1. Never or<1* per month 2. 1 * per month 3. 2-3 * per month 4. 1 * per week 5. 2-3 * per week 6. 4-6 * per week 7. Every day 8. More onetime per day |  |

| Thinking back on the last three months, please tell me how often you consumed each of the following items. You can tell me in terms of times per day, per week or per month. **ENTER 0 IF NEVER CONSUMED THE ITEM(Fruits)** | | | | | |
| --- | --- | --- | --- | --- | --- |
| K23.Banana | 1. Never or<1* per month 2. 1 * per month 3. 2-3 * per month 4. 1 * per week 5. 2-3 * per week 6. 4-6 * per week 7. Every day 8. More onetime per day |  | K24.Orange | 1. Never or<1* per month 2. 1 * per month 3. 2-3 * per month 4. 1 * per week 5. 2-3 * per week 6. 4-6 * per week 7. Every day 8. More onetime per day |  |
| K25. Mango | 1. Never or<1* per month 2. 1 * per month 3. 2-3 * per month 4. 1 * per week 5. 2-3 * per week 6. 4-6 * per week 7. Every day 8. More onetime per day |  | K26.Avocado | 1. Never or<1* per month 2. 1 * per month 3. 2-3 * per month 4. 1 * per week 5. 2-3 * per week 6. 4-6 * per week 7. Every day 8. More onetime per day |  |
| K27. Guava(zeyituna) | 1. Never or<1* per month 2. 1 * per month 3. 2-3 * per month 4. 1 * per week 5. 2-3 * per week 6. 4-6 * per week 7. Every day 8. More onetime per day |  | K28.Papaya | 1. Never or<1* per month 2. 1 * per month 3. 2-3 * per month 4. 1 * per week 5. 2-3 * per week 6. 4-6 * per week 7. Every day 8. More onetime per day |  |
| K29. Preem | 1. Never or<1* per month 2. 1 * per month 3. 2-3 * per month 4. 1 * per week 5. 2-3 * per week 6. 4-6 * per week 7. Every day 8. More onetime per day |  | K30.pin apple | 1. Never or<1* per month 2. 1 * per month 3. 2-3 * per month 4. 1 * per week 5. 2-3 * per week 6. 4-6 * per week 7. Every day 8. More onetime per day |  |

| Thinking back on the last three months, please tell me how often you consumed each of the following items. You can tell me in terms of times per day, per week or per month. **ENTER 0 IF NEVER CONSUMED THE ITEM(Meat and polutary)** | | | | | |
| --- | --- | --- | --- | --- | --- |
| K31. Beef | 1. Never or<1* per month 2. 1 * per month 3. 2-3 * per month 4. 1 * per week 5. 2-3 * per week 6. 4-6 * per week 7. Every day 8. More onetime per day |  | K32. Lamb | 1. Never or<1* per month 2. 1 * per month 3. 2-3 * per month 4. 1 * per week 5. 2-3 * per week 6. 4-6 * per week 7. Every day 8. More onetime per day |  |
| K33.Chicken meat (polutary) | 1. Never or<1* per month 2. 1 * per month 3. 2-3 * per month 4. 1 * per week 5. 2-3 * per week 6. 4-6 * per week 7. Every day 8. More onetime per day |  | K34. Goat meat | 1. Never or<1* per month 2. 1 * per month 3. 2-3 * per month 4. 1 * per week 5. 2-3 * per week 6. 4-6 * per week 7. Every day 8. More onetime per day |  |
| K35.Fish | 1. Never or<1* per month 2. 1 * per month 3. 2-3 * per month 4. 1 * per week 5. 2-3 * per week 6. 4-6 * per week 7. Every day 8. More onetime per day |  | K36. Egg | 1. Never or<1* per month 2. 1 * per month 3. 2-3 * per month 4. 1 * per week 5. 2-3 * per week 6. 4-6 * per week 7. Every day 8. More onetime per day |  |

| Thinking back on the last three months, please tell me how often you consumed each of the following items. You can tell me in terms of times per day, per week or per month. **ENTER 0 IF NEVER CONSUMED THE ITEM(Milk and dairy products)** | | | | | |
| --- | --- | --- | --- | --- | --- |
| K37. Milk (cow’s milk) | 1. Never or<1* per month 2. 1 * per month 3. 2-3 * per month 4. 1 * per week 5. 2-3 * per week 6. 4-6 * per week 7. Every day 8. More onetime per day |  | K38.Cheese | 1. Never or<1* per month 2. 1 * per month 3. 2-3 * per month 4. 1 * per week 5. 2-3 * per week 6. 4-6 * per week 7. Every day 8. More onetime per day |  |
| K39.Yoghurt | 1. Never or<1* per month 2. 1 * per month 3. 2-3 * per month 4. 1 * per week 5. 2-3 * per week 6. 4-6 * per week 7. Every day 8. More onetime per day |  | K40. Packed milk | 1. Never or<1* per month 2. 1 * per month 3. 2-3 * per month 4. 1 * per week 5. 2-3 * per week 6. 4-6 * per week 7. Every day 8. More onetime per day |  |

| Thinking back on the last three months, please tell me how often you consumed each of the following items. You can tell me in terms of times per day, per week or per month. **ENTER 0 IF NEVER CONSUMED THE ITEM(Fat and oils)** | | | | | |
| --- | --- | --- | --- | --- | --- |
| K41.Butter | 1. Never or<1* per month 2. 1 * per month 3. 2-3 * per month 4. 1 * per week 5. 2-3 * per week 6. 4-6 * per week 7. Every day 8. More onetime per day |  | K42.Oil /plant palm/saturated/yerega | 1. Never or<1* per month 2. 1 * per month 3. 2-3 * per month 4. 1 * per week 5. 2-3 * per week 6. 4-6 * per week 7. Every day 8. More onetime per day |  |

| Thinking back on the last three months, please tell me how often you consumed each of the following items. You can tell me in terms of times per day, per week or per month. **ENTER 0 IF NEVER CONSUMED THE ITEM(Sweets)** | | | | | |
| --- | --- | --- | --- | --- | --- |
| K43.Honey | 1. Never or<1* per month 2. 1 * per month 3. 2-3 * per month 4. 1 * per week 5. 2-3 * per week 6. 4-6 * per week 7. Every day 8. More onetime per day |  | K44.Sugar | 1. Never or<1* per month 2. 1 * per month 3. 2-3 * per month 4. 1 * per week 5. 2-3 * per week 6. 4-6 * per week 7. Every day 8. More onetime per day |  |
| K45.Soft drink (mirinda,pepsi,fanta,coca) | 1. Never or<1* per month 2. 1 * per month 3. 2-3 * per month 4. 1 * per week 5. 2-3 * per week 6. 4-6 * per week 7. Every day 8. More onetime per day |  | K46.Cake (biscuits) | 1. Never or<1* per month 2. 1 * per month 3. 2-3 * per month 4. 1 * per week 5. 2-3 * per week 6. 4-6 * per week 7. Every day 8. More onetime per day |  |

| Thinking back on the last three months, please tell me how often you consumed each of the following items. You can tell me in terms of times per day, per week or per month. **ENTER 0 IF NEVER CONSUMED THE ITEM(Fast foods)** | | | | | |
| --- | --- | --- | --- | --- | --- |
| K47.Burger | 1. Never or<1* per month 2. 1 * per month 3. 2-3 * per month 4. 1 * per week 5. 2-3 * per week 6. 4-6 * per week 7. Every day 8. More onetime per day |  | K48.Pizza | 1. Never or<1* per month 2. 1 * per month 3. 2-3 * per month 4. 1 * per week 5. 2-3 * per week 6. 4-6 * per week 7. Every day 8. More onetime per day |  |
| K49.French fries (chips) | 1. Never or<1* per month 2. 1 * per month 3. 2-3 * per month 4. 1 * per week 5. 2-3 * per week 6. 4-6 * per week 7. Every day 8. More onetime per day |  | K50.Sambusa | 1. Never or<1* per month 2. 1 * per month 3. 2-3 * per month 4. 1 * per week 5. 2-3 * per week 6. 4-6 * per week 7. Every day 8. More onetime per day |  |

|  | **Section 3: Household food security,** | | |
| --- | --- | --- | --- |
| K51 | In the past four weeks, did you worry that your household would not have enough food? | 1=Yes 0=No |  |
| K52 | If yes, how often did this happen? | 1 = Rarely (once\twice in the past 4 weeks)  2 = Sometimes (3 to 10 times in the past four weeks)  3 = Often (more than 10 times in the past four weeks) |  |
| K53 | In the past four weeks, were you or any household member not able to eat the kinds of foods you preferred because of a lack of resources? | 1=Yes 0=No |  |
| K54 | If yes, how often did this happen? | 1 = Rarely (once\twice in the past 4 weeks)  2 = Sometimes (3 to 10 times in the past four weeks)  3 = Often (more than 10 times in the past four weeks) |  |
| K54 | In the past four weeks, did you or any household member have to eat a limited variety of foods due to a lack of resources? | 1=Yes 0=No |  |
| K55 | If yes, how often did this happen? | 1 = Rarely (once\twice in the past 4 weeks)  2 = Sometimes (3 to 10 times in the past four weeks)  3 = Often (more than 10 times in the past four weeks) |  |
| K56 | In the past four weeks, did you or any household member have (asked) to eat some foods that you really did not want to eat because of a lack of resources to obtain other types of food? | 1=Yes 0=No |  |
| K57 | If yes, how often did this happen? | 1 = Rarely (once\twice in the past 4 weeks)  2 = Sometimes (3 to 10 times in the past four weeks)  3 = Often (more than 10 times in the past four weeks) |  |
| K58 | In the past four weeks, did you or any household member have to eat a smaller meal than you felt you needed because there was not enough food? | 1=Yes 0=No |  |
| K59 | If yes, how often did this happen? | 1 = Rarely (once\twice in the past 4 weeks)  2 = Sometimes (3 to 10 times in the past four weeks)  3 = Often (more than 10 times in the past four weeks) |  |
| K60 | In the past four weeks, did you or any household member have to eat fewer meals in a day because there was not enough food? | 1=Yes 0=No |  |
| K61 | If yes, how often did this happen? | 1 = Rarely (once\twice in the past 4 weeks)  2 = Sometimes (3 to 10 times in the past four weeks)  3 = Often (more than 10 times in the past four weeks) |  |
| K62 | In the past four weeks, were you or any household member not able to eat the kinds of foods you preferred because of a lack of resources? | 1=Yes 0=No |  |
| K63 | If yes, how often did this happen? | 1 = Rarely (once\twice in the past 4 weeks)  2 = Sometimes (3 to 10 times in the past four weeks)  3 = Often (more than 10 times in the past four weeks) |  |
| K64 | In the past four weeks, did you or any household member go to sleep at night hungry because there was not enough food? | 1=Yes 0=No |  |
| K65 | If yes, how often did this happen? | 1 = Rarely (once\twice in the past 4 weeks)  2 = Sometimes (3 to 10 times in the past four weeks)  3 = Often (more than 10 times in the past four weeks) |  |
| K66 | In the past four weeks, did you or any household member go a whole day and night without eating anything because there was not enough food? | 1=Yes 0=No |  |
| K67 | If yes, how often did this happen? | 1 = Rarely (once\twice in the past 4 weeks)  2 = Sometimes (3 to 10 times in the past four weeks)  3 = Often (more than 10 times in the past four weeks) |  |

|  | **Section 4: Water Source Use & Hygiene** | |  |
| --- | --- | --- | --- |
| K68 | What is your household’s most commonly used source of drinking water? | 1=Piped water, 2=Public tap, 3=Tube well or borehole, 4=Protected well or spring, 5=Unprotected well or spring, 6=Rainwater, 7=River or pond, 8=Bottled, 9=Other (specify_______) 98 = Don’t know |  |
| K69 | What is the water source for the child at school? | 1=Piped water, 2=Public tap, 3=Tube well or borehole, 4=Protected well or spring, 5=Unprotected well or spring, 6=Rainwater, 7=River or pond, 8=Bottled, 9=Other (specify_______) 98 = Don’t know |  |
| K70 | Do you do anything to your household water before drinking it? | 1=Do nothing, 2= Boiling, 3=Use traditional herbs, 4=Use chemicals (water guard, liquid, Wuha Agar/Bishangari), 5=Filter/sieve, 6=Decant, 7=Other (specify___________) 98 = Don’t know |  |
| K71 | Do you store drinking water separately from your other household water? | 1= Yes  0= No  98 = Don’t know |  |
| K72 | Where do you store your household drinking water? | 1=Traditional pot with cover, 2=Traditional pot without cover, 3=Plastic jerry can with cover, 4=Plastic jerry can without cover, 5=Other (specify__________) 98 = Don’t know |  |
| K73 | How much water (in 20L jerry cans) does your household usually use in one day? | ______ 20L jerry cans or equivalent  98 = Don’t know |  |
| K74 | Do you have a habit of hand washing before meal | 1.Yes  2.No |  |
| K75 | If yes, is that with________________ | 1.Water only  2.Soap and water |  |
| K76 | Do you have a habit of hand washing after soil contact | 1.Yes  2.No |  |
| K77 | If yes, is that with________________ | 1.Water only  2.Soap and water |  |
| K78 | Do you have a habit of hand washing after toilet use | 1.Yes  2.No |  |
| K79 | If yes, is that with________________ | 1.Water only  2.Soap and water |  |
| K80 | Do you have a habit of washing fruits and vegetables before eating | 1.Yes  2.No |  |
| K81 | Latrine availability in the school | 1.Yes  2.No |  |
| K82 | Do you walk on barefoot | 1.Yes  2.No |  |
| K82a | Do you a habit of Swimming/basing in the lake/rever etc | 1.yes  2. no |  |

|  | **Section 5: Health related factors** | | |
| --- | --- | --- | --- |
| K83 | Does your child have illness during the last two weeks? | 1.Yes  2.No |  |
| K84 | If yes does your child have constipation/diarrhea in the past two weeks? | 1.Yes  2.No |  |
| K85 | Does your child have cough in the past two weeks? | 1.Yes  2.No |  |
| K86 | Does your child have bloating in the past two weeks? | 1.Yes  2.No |  |
| K87 | Does your child have abdominal pain in the past two week? | 1.Yes  2.No |  |
| K88 | Does your child have less appetite/anoxia in the past two weeks due to the illness? | 1.Yes  2.No |  |
| K89 | Does your child have nausea /vomiting in the past two weeks? | 1.yes  2.No |  |
| K90 | Did your parents, teachers or health professionals gave you deworming pills? | 1.Yes  2. No  3. Don`t know |  |
| K91 | If yes, when was the last time you took the pills | ______ |  |

|  | **Section 6: Nutritional status of the CHILD** | |
| --- | --- | --- |
| 6.1.1 | MUAC |  |
|  | Weight |  |
|  | Height |  |
|  | BMI |  |
| 6.1.2 | Delivery methods | |
|  | 1.Vaginal Delivery |  |
|  | 2. CS |  |
| 6.1.3 | Child milk type | |
|  | 1.Brest Milk |  |
|  | 2.Formula Milk |  |
