# Supplementary material for: Prevalence, infection intensity, and risk factors of soil-transmitted helminthiasis and intestinal schistosomiasis among schoolchildren in Southern Ethiopia
Source: PLoS Negl Trop Dis. 2026 Mar 18;20(3):e0014115. doi: 10.1371/journal.pntd.0014115 (PMC13016474; doi:10.1371/journal.pntd.0014115)
Supplement: S3 Table — (DOCX) [file pntd.0014115.s003.docx]

| የትምህርት ቤት ስም  ______________________ | የልጅ ስም  _________________________ | መለያ ቁጥር  _______________________ |
| --- | --- | --- |
| Grade  ______________________ | የጠያቂው ስም  _________________________ | የቃለ መጠይቁ ቀን (ቀን/ወር/ዓመት)  ________________________ |

| **ክፍል አንድ በሶሺዮ-ዲሞግራፊግ ክፍል** ማህበራዊና የስነሕዝብ ባህሪያት  **( Socio-demographic part)** | | | ምላሽ | ኮድ/መለያ |
| --- | --- | --- | --- | --- |
| እድሜ (በአመት) | | _______ዓመት |  | A1 |
| ጾታ | | ወንድ  ሴት | 1  2 | A2 |
| ሃይማኖት | | ኦርቶዶክስ  ሙስሊም  ፕሮቴስታንት  ካቶሊክ  ሌላ (ይግለጹ)፣_________________________________ | 1  2  3  4 | A3 |
| መኖሪያ | | ከተማ  ከፊል ከተማ  ገጠር | 1  2  3 | A4 |
| የእናትየው የትምህርት ደረጃ | | መደበኛ ትምህርት አልተማረም  መጀመሪያና መለስተኛ ደረጃ  ሁለተኛ ደረጃ  ኮሌጅ እና ከዚያ በላይ | 1  2  3  4 | A6 |
| የአባት የትምህርት ደረጃ | | መደበኛ ትምህርት አልተማረም  መጀመሪያና መለስተኛ ደረጃ  ሁለተኛ ደረጃ  ኮሌጅ እና ከዚያ በላይ | 1  2  3  4 | A7 |
| ከፍተኛው የቤተሰብ የትምህርት ደረጃ ምን ያህል ነው? | | መጀመሪያና መለስተኛ(1-8)  ሁለተኛ ደረጃ (9-12)  ዲፕሎማ እና ከዚያ በላይ | 1  2  3 | A9 |
| የእናት የስራ ሁኔታ? | | የቤት እመቤት  የመንግስት ሰራተኛ  የግል ንግድ  ተማሪ  ሌሎች ይገልጻሉ............ | 1  2  3  4  5 | A10 |
| የአባት የስራ ሁኔታ? | | መንግስት ሰራተኛ  የቀን ሰራተኛ  የግል ንግድ  ተማሪ  ሌሎች __________ ይገልጻሉ | 1  2  3  4  5 | A11 |
| የሕፃኑ የኑሮ ሁኔታ | | ከወላጆች/አጋሮች ጋር መኖር  ከዘመድ ጋር መኖር  በኪራይ ቤት ውስጥ ከጓደኞች ጋር መኖር  በተከራይ ቤት ውስጥ ብቻውን መኖር  ሌሎች (ይግለጹ)_______ | 1  2  3  4  5 | A12 |
| አንተን/አንችን ጨምሮ የቤተሰብ ብዛት? | | _______ |  | A13 |
| **ክፍል ሁለት መጠያቂ የሀብት መረጃ ጠቋሚ** | | | ምላሽ | ኮድ |
| የእርስዎ ቤተሰብ የሚከተሉት ንብረቶች አሉት? | የሚሰራ ሬዲዮ/ቴፕ መቅጃ/ሲዲ ማጫወቻ | አዎ  አይ | 1  0 | A16 |
|  | የሚሰራ ቴሌቪዥን | አዎ  አይ | 1  0 | A17 |
|  | የጋዝ ምድጃ | አዎ  አይ | 1  0 | A18 |
|  | የኤሌክትሪክ ምድጃ | አዎ  አይ | 1  0 | A19 |
|  | ሞተር ብስክሌት | አዎ  አይ | 1  0 | A20 |
|  | ጋሪ | አዎ  አይ | 1  0 | A21 |
|  | ሰዓት (የእጅ/የግድግዳ) | አዎ  አይ | 1  0 | A22 |
|  | ሞባይል ስልክ | አዎ  አይ | 1  0 | A23 |
|  | ብስክሌት | አዎ  አይ | 1  0 | A24 |
|  | ሶፋ | አዎ  አይ | 1  0 | A25 |
|  | ስፖንጅ /ፍራሽ | አዎ  አይ | 1  0 | A26 |
|  | ወንበር/ ዱካ | አዎ  አይ | 1  0 | A27 |
|  | ጀነሬተር / የፀሐይ | አዎ  አይ | 1  0 | A28 |
|  | መፍጫ | አዎ  አይ | 1  0 | A29 |
|  | መኪና | አዎ  አይ | 1  0 | A30 |
|  | ማቀዝቀዣ | አዎ  አይ | 1  0 | A31 |
|  | ባጃጅ | አዎ  አይ | 1  0 | A32 |
|  | የራሱ ቤት ነው | አዎ  አይ | 1  0 | A33 |
|  | የቤት ዓይነት | ዘመናዊ የጣራ ቆርቆሮ  ባህላዊ/ጎጆ | 1  0 | A34 |
|  | ዘመናዊ ከሆነ የግድግዳ ዓይነት? | የሲሚንቶ ግድግዳ  የአፈር ግድግዳ | 1  0 | A35 |

**የምግብ ድግግሞሽ ጠያቂ**

መመሪያ**-** ውድ ምላሽ ሰጭ እባካችሁ ባለፉት ሶስት ወራት ውስጥ የበሉትን ምግብ እና መጠጦችን ለማስታወስ ጥቂት ጊዜ ይውሰዱ። እነዚህ ከእርስዎ የዕለት ተዕለት የምግብ አይነቶች አጠቃቀም እና እንዲሁም ከቤት ውጭ ከሚጠቀሙት ምግቦች ጋር ይዛመዳሉ፣ ለምሳሌ በእረፍት ጊዜ፣ ከቤት ውጭ ከጓደኞችዎ ጋር እና በትምህርት ቤት።የምግቡን አይነት ከበላህ ባለፉት ሶስት ወራት ውስጥ ምን ያህል ጊዜ እንደበላህ ትነግረኛለህ።

| ያለፉትን ሶስት ወራት መለስ ብልህ አስብ፣ እባኮትን የሚከተሉትን የምግብ አይነቶች ምን ያህል ጊዜ እንደወሰድክ ንገረኝ።  በቀን ፣በሳምንት ወይም በወር ጊዜዎች ሊነግሩኝ ይችላሉ።  በጭራሽ ካልበሉ 0 አስገቡ (እህል እና ጥራጥሬ) | | | | | |
| --- | --- | --- | --- | --- | --- |
| K1. ጤፍ | 1. በጭራሽ ወይም<1* በወር  2. 1 * በወር  3. 2-3 * በወር  4. 1 * በሳምንት  5. 2-3 * በሳምንት  6. 4-6 * በሳምንት  7. በየቀኑ  8. በቀን ከአንድ በላይ |  | K2. በቆሎ | 1. በጭራሽ ወይም<1* በወር  2. 1 * በወር  3. 2-3 * በወር  4. 1 * በሳምንት  5. 2-3 * በሳምንት  6. 4-6 * በሳምንት  7. በየቀኑ  8. በቀን ከአንድ በላይ |  |
| K3. ገብስ | 1. በጭራሽ ወይም<1* በወር  2. 1 * በወር  3. 2-3 * በወር  4. 1 * በሳምንት  5. 2-3 * በሳምንት  6. 4-6 * በሳምንት  7. በየቀኑ  8. በቀን ከአንድ በላይ |  | K4. ስንዴ፣  ዳቦን ጨምሮ | 1. በጭራሽ ወይም<1* በወር  2. 1 * በወር  3. 2-3 * በወር  4. 1 * በሳምንት  5. 2-3 * በሳምንት  6. 4-6 * በሳምንት  7. በየቀኑ  8. በቀን ከአንድ በላይ |  |
| K5. ማሽላ | 1. በጭራሽ ወይም<1* በወር  2. 1 * በወር  3. 2-3 * በወር  4. 1 * በሳምንት  5. 2-3 * በሳምንት  6. 4-6 * በሳምንት  7. በየቀኑ  8. በቀን ከአንድ በላይ |  | K6. ሩዝ | 1. በጭራሽ ወይም<1* በወር  2. 1 * በወር  3. 2-3 * በወር  4. 1 * በሳምንት  5. 2-3 * በሳምንት  6. 4-6 * በሳምንት  7. በየቀኑ  8. በቀን ከአንድ በላይ |  |

| ያለፉትን ሶስት ወራት መለስ ብልህ አስብ፣ እባኮትን የሚከተሉትን የምግብ አይነቶች ምን ያህል ጊዜ እንደወሰድክ ንገረኝ።  በቀን ፣በሳምንት ወይም በወር ጊዜዎች ሊነግሩኝ ይችላሉ።  **በጭራሽ ካልበሉ 0 አስገቡ** | | | | | |
| --- | --- | --- | --- | --- | --- |
| K7. ፓስታ (ማካሮኒ) | 1. በጭራሽ ወይም<1* በወር  2. 1 * በወር  3. 2-3 * በወር  4. 1 * በሳምንት  5. 2-3 * በሳምንት  6. 4-6 * በሳምንት  7. በየቀኑ  8. በቀን ከአንድ በላይ |  | K8. የአጃ ቅንጨ(አጃ) | 1. በጭራሽ ወይም<1* በወር  2. 1 * በወር  3. 2-3 * በወር  4. 1 * በሳምንት  5. 2-3 * በሳምንት  6. 4-6 * በሳምንት  7. በየቀኑ  8. በቀን ከአንድ በላይ |  |
| K9. ምስር | 1. በጭራሽ ወይም<1* በወር  2. 1 * በወር  3. 2-3 * በወር  4. 1 * በሳምንት  5. 2-3 * በሳምንት  6. 4-6 * በሳምንት  7. በየቀኑ  8. በቀን ከአንድ በላይ |  | K10. በጭንቅ(Barely) (ገንፎ፣ poridge) | 1. በጭራሽ ወይም<1* በወር  2. 1 * በወር  3. 2-3 * በወር  4. 1 * በሳምንት  5. 2-3 * በሳምንት  6. 4-6 * በሳምንት  7. በየቀኑ  8. በቀን ከአንድ በላይ |  |
| K11. ሺንቤራ | 1. በጭራሽ ወይም<1* በወር  2. 1 * በወር  3. 2-3 * በወር  4. 1 * በሳምንት  5. 2-3 * በሳምንት  6. 4-6 * በሳምንት  7. በየቀኑ  8. በቀን ከአንድ በላይ |  | K12. ባቄላ | 1. በጭራሽ ወይም<1* በወር  2. 1 * በወር  3. 2-3 * በወር  4. 1 * በሳምንት  5. 2-3 * በሳምንት  6. 4-6 * በሳምንት  7. በየቀኑ  8. በቀን ከአንድ በላይ |  |

| ያለፉትን ሶስት ወራት መለስ ብልህ አስብ፣ እባኮትን የሚከተሉትን የምግብ አይነቶች ምን ያህል ጊዜ እንደወሰድክ ንገረኝ።  በቀን ፣በሳምንት ወይም በወር ጊዜዎች ሊነግሩኝ ይችላሉ።  **በጭራሽ ካልበሉ 0 አስገቡ (ስራ** ሥሮች እና ቱቦዎች**)** | | | | | |
| --- | --- | --- | --- | --- | --- |
| K13.ቆጮ | 1. በጭራሽ ወይም<1* በወር  2. 1 * በወር  3. 2-3 * በወር  4. 1 * በሳምንት  5. 2-3 * በሳምንት  6. 4-6 * በሳምንት  7. በየቀኑ  8. በቀን ከአንድ በላይ |  | K14. ስኮር ድንች | 1. በጭራሽ ወይም<1* በወር  2. 1 * በወር  3. 2-3 * በወር  4. 1 * በሳምንት  5. 2-3 * በሳምንት  6. 4-6 * በሳምንት  7. በየቀኑ  8. በቀን ከአንድ በላይ |  |
| K15. ካሮት | 1. በጭራሽ ወይም<1* በወር  2. 1 * በወር  3. 2-3 * በወር  4. 1 * በሳምንት  5. 2-3 * በሳምንት  6. 4-6 * በሳምንት  7. በየቀኑ  8. በቀን ከአንድ በላይ |  | K16. ድንች | 1. በጭራሽ ወይም<1* በወር  2. 1 * በወር  3. 2-3 * በወር  4. 1 * በሳምንት  5. 2-3 * በሳምንት  6. 4-6 * በሳምንት  7. በየቀኑ  8. በቀን ከአንድ በላይ |  |

| ያለፉትን ሶስት ወራት መለስ ብልህ አስብ፣ እባኮትን የሚከተሉትን የምግብ አይነቶች ምን ያህል ጊዜ እንደወሰድክ ንገረኝ።  በቀን ፣በሳምንት ወይም በወር ጊዜዎች ሊነግሩኝ ይችላሉ።  **በጭራሽ ካልበሉ 0 አስገቡ** **(**አትክልቶች**)** | | | | | |
| --- | --- | --- | --- | --- | --- |
| K17. ኮስጣ | 1. በጭራሽ ወይም<1* በወር  2. 1 * በወር  3. 2-3 * በወር  4. 1 * በሳምንት  5. 2-3 * በሳምንት  6. 4-6 * በሳምንት  7. በየቀኑ  8. በቀን ከአንድ በላይ |  | K18. ጎመን | 1. በጭራሽ ወይም<1* በወር  2. 1 * በወር  3. 2-3 * በወር  4. 1 * በሳምንት  5. 2-3 * በሳምንት  6. 4-6 * በሳምንት  7. በየቀኑ  8. በቀን ከአንድ በላይ |  |
| K19. ጥቁር ጎመን | 1. በጭራሽ ወይም<1* በወር  2. 1 * በወር  3. 2-3 * በወር  4. 1 * በሳምንት  5. 2-3 * በሳምንት  6. 4-6 * በሳምንት  7. በየቀኑ  8. በቀን ከአንድ በላይ |  | K20. ቲማቲም | 1. በጭራሽ ወይም<1* በወር  2. 1 * በወር  3. 2-3 * በወር  4. 1 * በሳምንት  5. 2-3 * በሳምንት  6. 4-6 * በሳምንት  7. በየቀኑ  8. በቀን ከአንድ በላይ |  |
| K21. ዱባ | 1. በጭራሽ ወይም<1* በወር  2. 1 * በወር  3. 2-3 * በወር  4. 1 * በሳምንት  5. 2-3 * በሳምንት  6. 4-6 * በሳምንት  7. በየቀኑ  8. በቀን ከአንድ በላይ |  | K22. ፎሶሊያ | 1. በጭራሽ ወይም<1* በወር  2. 1 * በወር  3. 2-3 * በወር  4. 1 * በሳምንት  5. 2-3 * በሳምንት  6. 4-6 * በሳምንት  7. በየቀኑ  8. በቀን ከአንድ በላይ |  |

| ያለፉትን ሶስት ወራት መለስ ብልህ አስብ፣ እባኮትን የሚከተሉትን የምግብ አይነቶች ምን ያህል ጊዜ እንደወሰድክ ንገረኝ።  በቀን ፣በሳምንት ወይም በወር ጊዜዎች ሊነግሩኝ ይችላሉ።  **በጭራሽ ካልበሉ 0 አስገቡ** **(**ፍራፍሬዎች**)** | | | | | |
| --- | --- | --- | --- | --- | --- |
| K23. ሙዝ | 1. በጭራሽ ወይም<1* በወር  2. 1 * በወር  3. 2-3 * በወር  4. 1 * በሳምንት  5. 2-3 * በሳምንት  6. 4-6 * በሳምንት  7. በየቀኑ  8. በቀን ከአንድ በላይ |  | K24. ብርቱካን | 1. በጭራሽ ወይም<1* በወር  2. 1 * በወር  3. 2-3 * በወር  4. 1 * በሳምንት  5. 2-3 * በሳምንት  6. 4-6 * በሳምንት  7. በየቀኑ  8. በቀን ከአንድ በላይ |  |
| K25. ማንጎ | 1. በጭራሽ ወይም<1* በወር  2. 1 * በወር  3. 2-3 * በወር  4. 1 * በሳምንት  5. 2-3 * በሳምንት  6. 4-6 * በሳምንት  7. በየቀኑ  8. በቀን ከአንድ በላይ |  | K26. አቮካዶ | 1. በጭራሽ ወይም<1* በወር  2. 1 * በወር  3. 2-3 * በወር  4. 1 * በሳምንት  5. 2-3 * በሳምንት  6. 4-6 * በሳምንት  7. በየቀኑ  8. በቀን ከአንድ በላይ |  |
| K27. ዘይቱና | 1. በጭራሽ ወይም<1* በወር  2. 1 * በወር  3. 2-3 * በወር  4. 1 * በሳምንት  5. 2-3 * በሳምንት  6. 4-6 * በሳምንት  7. በየቀኑ  8. በቀን ከአንድ በላይ |  | K28. ፓፓያ | 1. በጭራሽ ወይም<1* በወር  2. 1 * በወር  3. 2-3 * በወር  4. 1 * በሳምንት  5. 2-3 * በሳምንት  6. 4-6 * በሳምንት  7. በየቀኑ  8. በቀን ከአንድ በላይ |  |
| K29. ፕሪም | 1. በጭራሽ ወይም<1* በወር  2. 1 * በወር  3. 2-3 * በወር  4. 1 * በሳምንት  5. 2-3 * በሳምንት  6. 4-6 * በሳምንት  7. በየቀኑ  8. በቀን ከአንድ በላይ |  | K30.አናናስ | 1. በጭራሽ ወይም<1* በወር  2. 1 * በወር  3. 2-3 * በወር  4. 1 * በሳምንት  5. 2-3 * በሳምንት  6. 4-6 * በሳምንት  7. በየቀኑ  8. በቀን ከአንድ በላይ |  |

| ያለፉትን ሶስት ወራት መለስ ብልህ አስብ፣ እባኮትን የሚከተሉትን የምግብ አይነቶች ምን ያህል ጊዜ እንደወሰድክ ንገረኝ።  በቀን ፣በሳምንት ወይም በወር ጊዜዎች ሊነግሩኝ ይችላሉ።  **በጭራሽ ካልበሉ 0 አስገቡ** **(**ስጋ እና የዶሮ **ተዋጻዖ)** | | | | | |
| --- | --- | --- | --- | --- | --- |
| K31. የበሬ ሥጋ | 1. በጭራሽ ወይም<1* በወር  2. 1 * በወር  3. 2-3 * በወር  4. 1 * በሳምንት  5. 2-3 * በሳምንት  6. 4-6 * በሳምንት  7. በየቀኑ  8. በቀን ከአንድ በላይ |  | K32. በግ | 1. በጭራሽ ወይም<1* በወር  2. 1 * በወር  3. 2-3 * በወር  4. 1 * በሳምንት  5. 2-3 * በሳምንት  6. 4-6 * በሳምንት  7. በየቀኑ  8. በቀን ከአንድ በላይ |  |
| K33. የዶሮ ሥጋ | 1. በጭራሽ ወይም<1* በወር  2. 1 * በወር  3. 2-3 * በወር  4. 1 * በሳምንት  5. 2-3 * በሳምንት  6. 4-6 * በሳምንት  7. በየቀኑ  8. በቀን ከአንድ በላይ |  | K34. የፍየል ስጋ | 1. በጭራሽ ወይም<1* በወር  2. 1 * በወር  3. 2-3 * በወር  4. 1 * በሳምንት  5. 2-3 * በሳምንት  6. 4-6 * በሳምንት  7. በየቀኑ  8. በቀን ከአንድ በላይ |  |
| K35. ዓሳ | 1. በጭራሽ ወይም<1* በወር  2. 1 * በወር  3. 2-3 * በወር  4. 1 * በሳምንት  5. 2-3 * በሳምንት  6. 4-6 * በሳምንት  7. በየቀኑ  8. በቀን ከአንድ በላይ |  | K36. እንቁላል | 1. በጭራሽ ወይም<1* በወር  2. 1 * በወር  3. 2-3 * በወር  4. 1 * በሳምንት  5. 2-3 * በሳምንት  6. 4-6 * በሳምንት  7. በየቀኑ  8. በቀን ከአንድ በላይ |  |

| ያለፉትን ሶስት ወራት መለስ ብልህ አስብ፣ እባኮትን የሚከተሉትን የምግብ አይነቶች ምን ያህል ጊዜ እንደወሰድክ ንገረኝ።  በቀን ፣በሳምንት ወይም በወር ጊዜዎች ሊነግሩኝ ይችላሉ።  **በጭራሽ ካልበሉ 0 አስገቡ** **(**ወተት እና የወተት ተዋጽኦዎች**)** | | | | | |
| --- | --- | --- | --- | --- | --- |
| K37. ወተት (የላም ወተት) | 1. በጭራሽ ወይም<1* በወር  2. 1 * በወር  3. 2-3 * በወር  4. 1 * በሳምንት  5. 2-3 * በሳምንት  6. 4-6 * በሳምንት  7. በየቀኑ  8. በቀን ከአንድ በላይ |  | K38. አይብ | 1. በጭራሽ ወይም<1* በወር  2. 1 * በወር  3. 2-3 * በወር  4. 1 * በሳምንት  5. 2-3 * በሳምንት  6. 4-6 * በሳምንት  7. በየቀኑ  8. በቀን ከአንድ በላይ |  |
| K39. እርጎ | 1. በጭራሽ ወይም<1* በወር  2. 1 * በወር  3. 2-3 * በወር  4. 1 * በሳምንት  5. 2-3 * በሳምንት  6. 4-6 * በሳምንት  7. በየቀኑ  8. በቀን ከአንድ በላይ |  | K40. የታሸገ ወተት | 1. በጭራሽ ወይም<1* በወር  2. 1 * በወር  3. 2-3 * በወር  4. 1 * በሳምንት  5. 2-3 * በሳምንት  6. 4-6 * በሳምንት  7. በየቀኑ  8. በቀን ከአንድ በላይ |  |

| ያለፉትን ሶስት ወራት መለስ ብልህ አስብ፣ እባኮትን የሚከተሉትን የምግብ አይነቶች ምን ያህል ጊዜ እንደወሰድክ ንገረኝ።  በቀን ፣በሳምንት ወይም በወር ጊዜዎች ሊነግሩኝ ይችላሉ።  **በጭራሽ ካልበሉ 0 አስገቡ** **(**ስብ እና ዘይቶች**)** | | | | | |
| --- | --- | --- | --- | --- | --- |
| K41. ቅቤ | 1. በጭራሽ ወይም<1* በወር  2. 1 * በወር  3. 2-3 * በወር  4. 1 * በሳምንት  5. 2-3 * በሳምንት  6. 4-6 * በሳምንት  7. በየቀኑ  8. በቀን ከአንድ በላይ |  | K42. ዘይት /የተክል ፓልም/ የሳቹሬትድ/ የረጋ | 1. በጭራሽ ወይም<1* በወር  2. 1 * በወር  3. 2-3 * በወር  4. 1 * በሳምንት  5. 2-3 * በሳምንት  6. 4-6 * በሳምንት  7. በየቀኑ  8. በቀን ከአንድ በላይ |  |

| ያለፉትን ሶስት ወራት መለስ ብልህ አስብ፣ እባኮትን የሚከተሉትን የምግብ አይነቶች ምን ያህል ጊዜ እንደወሰድክ ንገረኝ።  በቀን ፣በሳምንት ወይም በወር ጊዜዎች ሊነግሩኝ ይችላሉ።  **በጭራሽ ካልበሉ 0 አስገቡ** **(**ጣፋጮች**)** | | | | | |
| --- | --- | --- | --- | --- | --- |
| K43. ማር | 1. በጭራሽ ወይም<1* በወር  2. 1 * በወር  3. 2-3 * በወር  4. 1 * በሳምንት  5. 2-3 * በሳምንት  6. 4-6 * በሳምንት  7. በየቀኑ  8. በቀን ከአንድ በላይ |  | K44. ስኳር | 1. በጭራሽ ወይም<1* በወር  2. 1 * በወር  3. 2-3 * በወር  4. 1 * በሳምንት  5. 2-3 * በሳምንት  6. 4-6 * በሳምንት  7. በየቀኑ  8. በቀን ከአንድ በላይ |  |
| K45. ለስላሳ መጠጥ (ሚሪንዳ፣ፔፕሲ፣ፋንታ፣ኮካ) | 1. በጭራሽ ወይም<1* በወር  2. 1 * በወር  3. 2-3 * በወር  4. 1 * በሳምንት  5. 2-3 * በሳምንት  6. 4-6 * በሳምንት  7. በየቀኑ  8. በቀን ከአንድ በላይ |  | K46. ኬክ (ብስኩት) | 1. በጭራሽ ወይም<1* በወር  2. 1 * በወር  3. 2-3 * በወር  4. 1 * በሳምንት  5. 2-3 * በሳምንት  6. 4-6 * በሳምንት  7. በየቀኑ  8. በቀን ከአንድ በላይ |  |

| ያለፉትን ሶስት ወራት መለስ ብልህ አስብ፣ እባኮትን የሚከተሉትን የምግብ አይነቶች ምን ያህል ጊዜ እንደወሰድክ ንገረኝ።  በቀን ፣በሳምንት ወይም በወር ጊዜዎች ሊነግሩኝ ይችላሉ።  **በጭራሽ ካልበሉ 0 አስገቡ** **(**ፈጣን ምግቦች**)** | | | | | |
| --- | --- | --- | --- | --- | --- |
| K47. በርገር | 1. በጭራሽ ወይም<1* በወር  2. 1 * በወር  3. 2-3 * በወር  4. 1 * በሳምንት  5. 2-3 * በሳምንት  6. 4-6 * በሳምንት  7. በየቀኑ  8. በቀን ከአንድ በላይ |  | K48. ፒዛ | 1. በጭራሽ ወይም<1* በወር  2. 1 * በወር  3. 2-3 * በወር  4. 1 * በሳምንት  5. 2-3 * በሳምንት  6. 4-6 * በሳምንት  7. በየቀኑ  8. በቀን ከአንድ በላይ |  |
| K49. ቺፕስ | 1. በጭራሽ ወይም<1* በወር  2. 1 * በወር  3. 2-3 * በወር  4. 1 * በሳምንት  5. 2-3 * በሳምንት  6. 4-6 * በሳምንት  7. በየቀኑ  8. በቀን ከአንድ በላይ |  | K50. ሳምቡሳ | 1. በጭራሽ ወይም<1* በወር  2. 1 * በወር  3. 2-3 * በወር  4. 1 * በሳምንት  5. 2-3 * በሳምንት  6. 4-6 * በሳምንት  7. በየቀኑ  8. በቀን ከአንድ በላይ |  |

|  | **ክፍል 3፡ የቤተሰብ ምግብ ዋስትና** | | |
| --- | --- | --- | --- |
| K51 | ባለፉት አራት ሳምንታት ውስጥ፣ የእርስዎ ቤተሰብ በቂ ምግብ አይኖረውም ብለው ተጨንቀው ነበር? | 1=አዎ 0=አይደለም |  |
| K52 | አዎ ከሆነ፣ ይህ ምን ያህል ጊዜ ተከሰተ? | 1 = አልፎ አልፎ (ባለፉት 4 ሳምንታት ውስጥ አንድ ጊዜ\2 ጊዜ)  2 = አንዳንድ ጊዜ (ባለፉት አራት ሳምንታት ከ3 እስከ 10 ጊዜ)  3 = ብዙ ጊዜ (ባለፉት አራት ሳምንታት ውስጥ ከ 10 ጊዜ በላይ)) |  |
| K53 | ባለፉት አራት ሳምንታት ውስጥ እርስዎ ወይም ማንኛውም የቤተሰብ አባል በግብአት እጦት ምክንያት የመረጡትን አይነት ምግብ መመገብ አልቻላችሁም? | 1=አዎ 0=አይደለም |  |
| K54 | አዎ ከሆነ፣ ይህ ምን ያህል ጊዜ ተከሰተ? | 1 = አልፎ አልፎ (ባለፉት 4 ሳምንታት ውስጥ አንድ ጊዜ\2 ጊዜ)  2 = አንዳንድ ጊዜ (ባለፉት አራት ሳምንታት ከ3 እስከ 10 ጊዜ)  3 = ብዙ ጊዜ (ባለፉት አራት ሳምንታት ውስጥ ከ 10 ጊዜ በላይ)) |  |
| K54 | ባለፉት አራት ሳምንታት ውስጥ እርስዎ ወይም ማንኛውም የቤተሰብ አባል በግብአት እጦት ምክንያት የተወሰነ አይነት ምግብ መመገብ ተገዳችሁል? | 1=አዎ 0=አይደለም |  |
| K55 | አዎ ከሆነ፣ ይህ ምን ያህል ጊዜ ተከሰተ? | 1 = አልፎ አልፎ (ባለፉት 4 ሳምንታት ውስጥ አንድ ጊዜ\2 ጊዜ)  2 = አንዳንድ ጊዜ (ባለፉት አራት ሳምንታት ከ3 እስከ 10 ጊዜ)  3 = ብዙ ጊዜ (ባለፉት አራት ሳምንታት ውስጥ ከ 10 ጊዜ በላይ) |  |
| K56 | ባለፉት አራት ሳምንታት ውስጥ፣ እርስዎ ወይም ማንኛውም የቤተሰብ አባል ሌሎች የምግብ አይነቶችን ለማግኘት በግብአት እጥረት ምክንያት ለመመገብ ያልፈለጋችሁትን አንዳንድ ምግቦችን እንድትበሉ ጠየቋችሁ? | 1=አዎ 0=አይደለም |  |
| K57 | አዎ ከሆነ፣ ይህ ምን ያህል ጊዜ ተከሰተ? | 1 = አልፎ አልፎ (ባለፉት 4 ሳምንታት ውስጥ አንድ ጊዜ\2 ጊዜ)  2 = አንዳንድ ጊዜ (ባለፉት አራት ሳምንታት ከ3 እስከ 10 ጊዜ)  3 = ብዙ ጊዜ (ባለፉት አራት ሳምንታት ውስጥ ከ 10 ጊዜ በላይ) |  |
| K58 | ባለፉት አራት ሳምንታት ውስጥ እርስዎ ወይም ማንኛውም የቤተሰብ አባል በቂ ምግብ ስለሌለ ከምትፈልጉት ያነሰ ምግብ መብላት ነበረባችሁ? | 1=አዎ 0=አይደለም |  |
| K59 | አዎ ከሆነ፣ ይህ ምን ያህል ጊዜ ተከሰተ? | 1 = አልፎ አልፎ (ባለፉት 4 ሳምንታት ውስጥ አንድ ጊዜ\2 ጊዜ)  2 = አንዳንድ ጊዜ (ባለፉት አራት ሳምንታት ከ3 እስከ 10 ጊዜ)  3 = ብዙ ጊዜ (ባለፉት አራት ሳምንታት ውስጥ ከ 10 ጊዜ በላይ) |  |
| K60 | ባለፉት አራት ሳምንታት ውስጥ እርስዎ ወይም ማንኛውም የቤተሰብ አባል በቂ ምግብ ስለሌለ በቀን ውስጥ ጥቂት ምግቦችን መመገብ ነበረብዎት? | 1=አዎ 0=አይደለም |  |
| K61 | አዎ ከሆነ፣ ይህ ምን ያህል ጊዜ ተከሰተ? | 1 = አልፎ አልፎ (ባለፉት 4 ሳምንታት ውስጥ አንድ ጊዜ\2 ጊዜ)  2 = አንዳንድ ጊዜ (ባለፉት አራት ሳምንታት ከ3 እስከ 10 ጊዜ)  3 = ብዙ ጊዜ (ባለፉት አራት ሳምንታት ውስጥ ከ 10 ጊዜ በላይ) |  |
| K62 | ባለፉት አራት ሳምንታት ውስጥ እርስዎ ወይም ማንኛውም የቤተሰብ አባል በግብአት እጦት ምክንያት የመረጡትን አይነት ምግብ መመገብ አልቻላችሁም? | 1=አዎ 0=አይደለም |  |
| K63 | አዎ ከሆነ፣ ይህ ምን ያህል ጊዜ ተከሰተ? | 1 = አልፎ አልፎ (ባለፉት 4 ሳምንታት ውስጥ አንድ ጊዜ\2 ጊዜ)  2 = አንዳንድ ጊዜ (ባለፉት አራት ሳምንታት ከ3 እስከ 10 ጊዜ)  3 = ብዙ ጊዜ (ባለፉት አራት ሳምንታት ውስጥ ከ 10 ጊዜ በላይ) |  |
| K64 | ባለፉት አራት ሳምንታት ውስጥ እርስዎ ወይም ማንኛውም የቤተሰብ አባል በቂ ምግብ ስለሌለ እየተራቡ ተኝተው ነበር? | 1=አዎ 0=አይደለም |  |
| K65 | አዎ ከሆነ፣ ይህ ምን ያህል ጊዜ ተከሰተ? | 1 = አልፎ አልፎ (ባለፉት 4 ሳምንታት ውስጥ አንድ ጊዜ\2 ጊዜ)  2 = አንዳንድ ጊዜ (ባለፉት አራት ሳምንታት ከ3 እስከ 10 ጊዜ)  3 = ብዙ ጊዜ (ባለፉት አራት ሳምንታት ውስጥ ከ 10 ጊዜ በላይ) |  |
| K66 | ባለፉት አራት ሳምንታት ውስጥ እርስዎ ወይም ማንኛውም የቤተሰብ አባል በቂ ምግብ ስለሌለ ምንም ሳይበሉ ቀኑን ሙሉ ዋሉ? | 1=አዎ 0=አይደለም |  |
| K67 | አዎ ከሆነ፣ ይህ ምን ያህል ጊዜ ተከሰተ? | 1 = አልፎ አልፎ (ባለፉት 4 ሳምንታት ውስጥ አንድ ጊዜ\2 ጊዜ)  2 = አንዳንድ ጊዜ (ባለፉት አራት ሳምንታት ከ3 እስከ 10 ጊዜ)  3 = ብዙ ጊዜ (ባለፉት አራት ሳምንታት ውስጥ ከ 10 ጊዜ በላይ) |  |

|  | **ክፍል 4፡ የውሃ ምንጭ አጠቃቀም እና ንፅህና** | |  |
| --- | --- | --- | --- |
| K68 | የእርስዎ የመጠጥ ውሃ ምንጭ ምንድነው? | 1=የቧንቧ ውሃ፣ 2=የህዝብ ቧንቧ፣ 3=የቱቦ ጉድጓድ ወይም ጉድጓድ፣ 4=ጉድጓድ ወይም ምንጭ፣ 5=ያልተጠበቀ ጉድጓድ ወይም ምንጭ፣ 6=የዝናብ ውሃ፣ 7=ወንዝ ወይም ኩሬ፣ 8=የታሸገ፣ 9=ሌላ (ይግለጹ_______) 98=አላውቅም |  |
| K69 | በትምህርት ቤት ዉስጥ ለልጆች የውኃ ምንጭ ምንድን ነው? | 1=የቧንቧ ውሃ፣ 2=የህዝብ ቧንቧ፣ 3=የቱቦ ጉድጓድ ወይም ጉድጓድ፣ 4=ጉድጓድ ወይም ምንጭ፣ 5=ያልተጠበቀ ጉድጓድ ወይም ምንጭ፣ 6=የዝናብ ውሃ፣ 7=ወንዝ ወይም ኩሬ፣ 8= የታሸገ፣ 9=ሌላ (ይግለጹ_______) 98=አላውቅም |  |
| K70 | የመጠጥ ውሃ ከመጠቀሞ በፊት በቤትዎ ዉስጥ ምን ነገር ያደርጋሉ? | 1= ምንም አላድርግም፣ 2= መፍላት፣ 3= ባህላዊ እፅዋትን መጠቀም፣ 4= ​​ኬሚካሎችን ( ፈሳሽ፣ የውሃ አጋር/ቢሻንጋሪ)፣ 5=ማጣራት 6=ማስተካከያ፣ 7=ሌላ (ይግለጹ___________) 98=አላውቅም |  |
| K71 | የመጠጥ ውሃ ከሌላው የቤትዎ ውሃ ለይተው ያከማቻሉ? | 1= አዎ  0= አይ  98 = አላውቅም |  |
| K72 | የቤተሰብዎን የመጠጥ ውሃ ምን ዉስጥ ነው የሚያከማቹት? | 1=የባህላዊ ማሰሮ በክዳን፣ 2=የባህላዊ ማሰሮ ያለ ክዳን ፣ 3=የላስቲክ ጀሪካን ከክዳን ጋር፣ 4=የላስቲክ ጀሪካን ያለ ክዳን፣ 5=ሌላ (ይግለጹ__________) 98 = አያውቅም። |  |
| K73 | ቤተሰብዎ በአንድ ቀን ውስጥ ምን ያህል ውሃ (በ20 ሊትር ጀሪካን) ይጠቀማል? | ______ 20ሊ ጀሪካን ወይም ተመጣጣኝ  98 = አያውቅም |  |
| K74 | ከምግብ በፊት እጅን የመታጠብ ልማድ አለህ | 1. አዎ  2.አይ |  |
| K75 | አዎ ከሆነ፣ በምንድ ነው________________ | 1. ውሃ ብቻ  2. ሳሙና እና ውሃ |  |
| K76 | ከአፈር ንክኪ በኋላ እጅን የመታጠብ ልምድ አለ? | 1. አዎ  2.አይ |  |
| K77 | አዎ ከሆነ፣ በምንድ ነው________________ | 1. ውሃ ብቻ  2. ሳሙና እና ውሃ |  |
| K78 | ሽንት ቤት ከተጠቀሙ በኋላ እጅን የመታጠብ ልምድ አለ? | 1. አዎ  2.አይ |  |
| K79 | አዎ ከሆነ፣ በምንድ ነው________________ | 1. ውሃ ብቻ  2. ሳሙና እና ውሃ |  |
| K80 | አትክልትና ፍራፍሬ ከመብላታችሁ በፊት የማጠብ ልምድ አለ? | 1. አዎ  2.አይ |  |
| K81 | በትምህርት ቤት ውስጥ የመጸዳጃ ቤት አለ? | 1. አዎ  2.አይ |  |
| K82 | በባዶ እግረኛ ትሄዳለህ/ሽ? | 1. አዎ  2.አይ |  |

|  | **ክፍል 5፡ ከጤና ጋር የተያያዙ ምክንያቶች** | | |
| --- | --- | --- | --- |
| K83 | ልጅዎ ባለፉት ሁለት ሳምንታት ውስጥ ህመም አለበት? | 1. አዎ  2.አይ |  |
| K84 | አዎ ከሆነ ልጅዎ ባለፉት ሁለት ሳምንታት ውስጥ የሆድ ድርቀት/ተቅማጥ አለው? | 1. አዎ  2.አይ |  |
| K85 | ልጅዎ ባለፉት ሁለት ሳምንታት ውስጥ ሳል አለበት? | 1. አዎ  2.አይ |  |
| K86 | ልጅዎ ባለፉት ሁለት ሳምንታት ውስጥ ሆድ መነፋት አለበት? | 1. አዎ  2.አይ |  |
| K87 | ልጅዎ ባለፉት ሁለት ሳምንታት ውስጥ የሆድ ህመም አለበት? | 1. አዎ  2.አይ |  |
| K88 | ለፉት ሁለት ሳምንታት ውስጥ ልጅዎ በህመም ምክንያት ያነሰ የምግብ ፍላጎት/አኖክሲያ አለ ወይ? | 1. አዎ  2.አይ |  |
| K89 | ባለፉት ሁለት ሳምንታት ውስጥ ልጅዎ ማቅለሽለሽ / ማስታወክ አለበት? | 1. አዎ  2.አይ |  |
| K90 | ወላጆችህ፣ አስተማሪዎችህ ወይም የጤና ባለሙያዎች የሆድ ትላትል መከላከያ መድሃኒቶችን ሰጥተውህ ነበር? | 1. አዎ  2. አይ  3. አላውቅም |  |
| K91 | አዎ ከሆነ፣ ክኒኖችን ለመጨረሻ ጊዜ የወሰዱት መቼ ነበር? | ______ |  |

|  | ክፍል 6፡ የልጁ የስነምግብ ሁኔታ  **(Nutritional status of the CHILD)** | | |
| --- | --- | --- | --- |
| 6.1.1 | የመካከለኛው የላይኛው ክንድ ዙሪያ  (MUAC) | |  |
|  | ክብደት | |  |
|  | ቁመት | |  |
|  | የሰውነት ዉፍረት መረጃ ጠቋሚ  (BMI) | |  |
| 6.1.2 | የወሊድ አይነት(Delivery methods ) | | |
|  | 1.በመሃጸን(Vaginal Delivery) |  | |
|  | 2.በቀዶ ጥገና(Cesarean section CS |  | |
| 6.1.3 | የህጻን የወተት አይነት(Child milk type) | | |
|  | 1.የጡት ወተት(Brest Milk) |  | |
|  | 2.የጣሳ ወተት(Formula Milk) |  | |
|  |  | |  |
|  |  | |  |
|  |  | |  |
|  |  | |  |
|  |  | |  |
|  |  | |  |
|  |  | |  |
|  |  | |  |
|  |  | |  |
