# Supplementary material for: Prevalence, infection intensity, and risk factors of soil-transmitted helminthiasis and intestinal schistosomiasis among schoolchildren in Southern Ethiopia
Source: PLoS Negl Trop Dis. 2026 Mar 18;20(3):e0014115. doi: 10.1371/journal.pntd.0014115 (PMC13016474; doi:10.1371/journal.pntd.0014115)
Supplement: Attachment — Submitted filename: PNTD-D-25-01634_reviewer Ms.pdf [file pntd.0014115.s005.pdf]

PLOS Neglected Tropical Diseases

Prevalence and intensity of soil-transmitted helminthiasis and associated factors among school children in Hawella Tula Rural district, Hawassa, Sidama Regional State, Ethiopia

--Manuscript Draft--

**Manuscript Number:**	PNTD-D-25-01634
**Full Title:**	Prevalence and intensity of soil-transmitted helminthiasis and associated factors among school children in Hawella Tula Rural district, Hawassa, Sidama Regional State, Ethiopia
**Short Title:**	Prevalence and intensity of soil-transmitted helminthiasis and associated factors
**Article Type:**	Research Article
**Keywords:**	: Prevalence, Intensity of STH, Schistosoma, Factors; School children, Southern, Ethiopia
**Abstract:**	Background: Soil-transmitted helminthiasis (STH) is widespread and prevalent in tropical regions attributed to poor sanitation and limited healthcare access, resulting from overall well-being, developmental and cognitive impairments, particularly among children. This study aimed to assess the prevalence of STH and associated factors among schoolchildren in the Sidama Regional State of Ethiopia. The findings warrant and indicate the the urgent need for enhanced improved sanitation, health education and effective deworming initiatives.<br>Method: School-based cross-sectional study was conducted among 394 primary schoolchildren from June 2024 to December 2024. Data were collected using an interviewer-administered questionnaire. Infection with the parasite was diagnosed using Kato–Katz microscopic examination. Bivariate and multivariable logistic regression was used to identify factors associated with the STH infection.<br>Results: The overall prevalence of STH is 168 (42.6%) with a 95% CI (37.8-47.6%). The most prevalence parasite was Ascaris lumbricoides (n=115; 29.2%), followed by Trichuris trichiura (n=70; 17.8%), Hookworm spp (n=20; 5.1%), and Schistosomes (n=13; 3.3%). Schoolchildren with habits of eating unwashed fruits/vegetables AOR of 2.89 (95% CI: 1.73–4.85), swam or bathed in streams/lakes AOR of 2.23 (95% CI: 1.31–3.79), lacked deworming treatment AOR of 2.12 (95% CI: 1.26–3.56), were exposed to STHs including (A. lumbricoides, and T. trichiura).<br>Conclusion: Over one-third of schoolchildren had STH and Schistosoma. Factors that predict STH and Schistosoma include poor hygiene, lack of deworming, contaminated water, and; this implies the need for better hygiene, health education and deworming programs.<br><br>Keywords : Prevalence, Intensity of STH, Schistosoma, Factors; School children, Southern, Ethiopia
**Additional Information:**	
**Question**	**Response**
**Government Employee**<br><br>Are you or any of the contributing authors an employee of the United States government?<br><br>Manuscripts authored by one or more US Government employees are not copyrighted, but are licensed under a CC0 Public Domain Dedication, which allows	No - No authors are employees of the U.S. government.

Powered by Editorial Manager® and ProduXion Manager® from Aries Systems Corporation

unlimited distribution and reuse of the article for any lawful purpose. This is a legal requirement for US Government employees.<br><br>This will be typeset if the manuscript is accepted for publication.	
**Financial Disclosure**<br><br>Enter a financial disclosure statement that describes the sources of funding for the work included in this submission. Review the submission guidelines for detailed requirements. View published research articles from *PLOS Neglected Tropical Diseases* for specific examples.<br><br>This statement is required for submission and **will appear in the published article** if the submission is accepted. Please make sure it is accurate.<br><br>**Funded studies**<br>Enter a statement with the following details:<br>• Initials of the authors who received each award<br>• Grant numbers awarded to each author<br>• The full name of each funder<br>• URL of each funder website<br>• Did the sponsors or funders play any role in the study design, data collection and analysis, decision to publish, or preparation of the manuscript?<br><br>Did you receive funding for this work?	The author(s) received no specific funding for this work.
**Competing Interests**<br><br>On behalf of all authors, disclose any competing interests that could be perceived to bias this work.<br><br>This statement will be typeset if the manuscript is accepted for publication.	The authors declare that they have no conflict of interests.

Review the instructions link below and PLOS NTDs' competing interests policy to determine what information must be disclosed at submission.	
**Data Availability**<br><br>Provide a **Data Availability Statement** in the box below. This statement should detail where the data used in this submission can be accessed. This statement will be typeset if the manuscript is accepted for publication.<br><br>Before publication, authors are required to make all data underlying their findings fully available, without restriction. Review our PLOS Data Policy page for detailed information on this policy. Instructions for writing your Data Availability statement can be accessed via the Instructions link below.	Raw data used for preparation of this manuscript can be obtained from corresponding author with resealable request.

Manuscript

Click here to access/download;Manuscript;Manuscript Prevalence and intensity of soil 012.docx

Prevalence and intensity of soil-transmitted helminthiasis and associated factors among school children in Hawella Tula Rural district, Hawassa, Sidama Regional State, Ethiopia

Fisseha Bonja Geleto[1,4*], Berhanu Erko[2], Musa Mohammed Ali[1] Bineyam Taye[3], Hagos Ashenafi[2]

[1] Hawassa University, College of Medicine and Health Sciences, School of Medical Laboratory Science, Hawassa Ethiopia

[2] Addis Ababa University, Aklilu Lemma Institute of Health Research, Center for Pathobiology, Addis Ababa, Ethiopia.

[3] Colgate University, USA Department of Biology, Colgate University, Hamilton, New York

[4]Department of Microbiology, Immunology and Parasitology, Collage of Health Sciences, Addis Ababa University, Addis Ababa, Ethiopia.

Abstract

Background: Soil-transmitted helminthiasis (STH) is widespread and prevalent in tropical regions due to poor sanitation and limited healthcare access. This study aimed to assess the prevalence of STH and associated factors among schoolchildren in the Sidama Regional State of Ethiopia. **Method:** School-based cross-sectional study was conducted among 394 primary schoolchildren from June to December 2024. Data were collected using an interviewer-administered questionnaire. Infection with the parasite was diagnosed using Kato–Katz

microscopic examination. Bivariate and multivariable logistic regression was used to identify factors associated with the STH infection.

Results: A total of 394 school children were included in the study. The overall prevalence of STH is 42.6% with a 95% CI (37.8-47.6%). The most prevalence parasite was *Ascaris lumbricoides* (29.2%), followed by *Trichuris trichiura* (17.8%), hookworms (20; 5.1%), and *Schistosom*a *mansoni*es (3.3%). Schoolchildren with habits of eating unwashed fruits/vegetables (AOR: 2.89 (95% CI: 1.73–4.85), swam or bathed in streams/lakes (AOR: 2.23 (95% CI: 1.31–3.79), lacked deworming treatment (AOR: 2.12 (95% CI: 1.26–3.56), were exposed to STHs.

Conclusion: Over one-third of schoolchildren were infected with STH and *Schistosoma mansoni*. Factors that predict STH and *Schistosoma mansoni* infection include poor hygiene, lack of deworming, contaminated water, and; this implies the need for better hygiene, health education and deworming programs.

***Keywords*:** Prevalence, Intensity of STH, *Schistosoma mansoni*, Factors; School children, Ethiopia

Background

Soil-transmitted helminthiasis (STH), caused by *Ascaris lumbricoides*, *Trichuris trichiura*, and hookworms, is a major public health concern in regions with poor sanitation and inadequate access to clean water (1). These parasitic worms are transmitted through eggs present in human feces, contaminating the soil, especially among school-aged children (2). Infected children often suffer from anemia, stunted growth, and impaired cognitive development, which can affect their academic performance and overall well-being (3, 4). Effective control measures include periodic deworming with safe and effective medicines, health education to raise awareness of the disease, and improved sanitation to reduce soil contamination and break the cycle of transmission (5).

Globally, 4.5 billion people are at risk of STH infections, 1.5 billion infected people or 24% of the world's population (6). It causes clinical morbidity in 450 million, with Ascariasis (1,000 million), hookworm (900 million), and Trichuriasis (500 million) being among the top ten most common infections worldwide, significantly impacting physical and intellectual growth in children (7). The highest burden of helminth infection occurs in sub-Saharan Africa, the Latin America, China and East Asia (8). It is estimated that over 270 million preschool children and over 600 million school children are living in areas where the parasites are widely transmitted and need treatment and preventive interventions (9). The estimated number of people known to be infected globally is 807-1,121 million with *Ascaris lumbricoides*, 604-795 million with *Trichuris trichiura*, 576-740 million with hookworm and 240 million people infected with schistosomiasis (8). In sub-Saharan Africa, about 198, 192, 173, and 162 million people are infected with hookworm, Schistosomiasis species, *A. lumbricoides* and *T. trichiura*, respectively (10).

However, Ethiopia is working towards SDGs targeting STH infections under Goal 3, aiming to reduce morbidity and improve health, especially in children, as part of NTD control (11, 12). The prevalence of STH in Ethiopia is 32.6% - 54% in South Ethiopia (13, 14), a relatively higher was prevalence was also recorded 67.7% in previous studies carried out in Hawassa Tula Sub-City, Southern Ethiopia (15). In addition to this, 31.2% in North-West Ethiopia (16) and 36.78% in Ethiopia (17) can be mentioned. According to the 2016 EDHS, only 57% of rural households and 97% of urban households have access to improved drinking water sources (18), while just 60% of homes have hand washing facilities. Alarmingly, 39% of rural and 7% of urban households lack toilet facilities (19). Furthermore, 82% of the population rely on unimproved sanitation, and 38.1 million people still practice open defecation (20). This study provides critical evidence on the prevalence and distribution of STHs and associated factors among schoolchildren in southern Ethiopia, highlighting a significant public health challenge and informing targeted interventions for clinicians, public health officials, and policymakers to reduce the burden of STH and improve child health (21).

Methods

Study setting and design

The study was conducted among school aged children's students in Sidama National Regional State, Southern Ethiopia. Hawassa city had 8 sub cities and located 272 Kms south of Addis Ababa in the Great Rift Valley in Ethiopia. According to the projected population of the sub city in 2023, the population of the sub city was estimated to be 135,793 out of which about 69,255 were adult 33,670 were children in the 5–18 years age group. The district has an agrarian economy focused on

coffee, maize, livestock, and some Khat production. The Hawela Tula sub city is home to three governmental primary schools and, 2 private schools with 11,134 students enrolled among 3 governmental schools for the 2024 academic year. The main staple food is Enset (false banana), and coffee and maize are key crops grown in the area (22). The study was carried out from June to December 2024.

Study population and Eligibility criteria

This institution-based cross-sectional study focused on primary schoolchildren (5–18 years) and their caregivers in Hawella Tula Rural district, Hawassa, Sidama Regional State, Ethiopia. The study included those who had lived in the area for at least six months and excluded children with recent anti-helminthic treatment or a history of intestinal parasitic treatment within two weeks, as well as caregivers unable to provide consent due to serious illness.

Study variables

Outcome variable: Soil-transmitted helminth (STH) infections

Exposure variables: Socio-demographic and economic factors (child's age, sex, residence, parental education, occupation, family size, and household wealth) were considered. Environmental factors (soil contamination, poor sanitation, open defecation, proximity to water bodies) were taken into account. Behavioral aspects (barefoot walking, swimming in contaminated water, poor hand hygiene, improper food handling, lack of health awareness) were recorded. Additionally, WASH-related characteristics (access to clean water, availability of latrines, hand washing facilities, proper waste disposal) were assessed.

Sample size and sampling procedures

The sample size (n) was calculated using the following single population proportion formula provided by (23) based on the assumption of (p), prevalence of STH 54% in schoolchildren reported from South Ethiopia (13), 95% Confidence interval (CI) (1.96), 5% margin of error (d), and adding 10% contingency.

$$n = (Z_{1-\alpha/2})^2 \frac{P[1-P]}{d^2} \quad n = 420$$

Therefore, the required sample size was calculated and obtained as 420.

For the selection of the study participants, the sampling frame prepared from available in the district and, primary schools were used. The sample was allocated to all available schools. Then the study participants proportionally allocated to each school based on the number of children from 7–18. Finally, 420 study participants were selected by using Microsoft-Excel -generated random number (lottery method).

Data collection tools and procedures

Data collection tools

Data were collected using pre-tested, structured, and translated questionnaires administered via interviews. Socio-demographic factors and other determinants were assessed, with a pretest on 5% of the study population conducted two weeks prior to the actual study. Five clinical nurses, supervised by a BSc nurse and investigators, collected the data after two days of training and competency assessments.

Parasitological Examination

Stool samples were collected in clean, labeled plastic containers and examined using the Kato-Katz thick smear technique (24). A portion of the fecal sample was taken with a wooden spatula, sieved through a nylon screen, and transferred to a template on a microscope slide. The template hole was filled with the sieved sample, leveled, and covered with malachite green-glycerin-soaked cellophane squares. The slides were then examined under a microscope for STH eggs. a Kato-Katz thick smear was prepared from each sample and analyzed under a light microscope for hookworm eggs between 30 and 60 minutes after slide preparation (24). Egg counts were recorded as eggs per gram (EPG) by averaging counts from two slides and multiplying by a conversion factor of 24. Infection intensity was categorized as light, moderate, or heavy based on WHO criteria. For quality control, duplicate slides were prepared for each sample and examined by two different technicians. Negative samples were rechecked the same day to ensure accuracy (25).

Data Analysis

Data was analyzed using SPSS version 23. Descriptive analysis including frequency distribution and the percentage was made to determine the prevalence of STH, to describe socio-economic and demographic and other determinants. Bivariate logistic regression analysis was conducted for crude odds ratio (COR) and all factors with a p-value <0.25 were considered as a candidate to a multivariable logistic regression to control confounding effects. The Hosmer -Leme_show goodness-of-fit statistic was used to assess whether the necessary assumptions for the application of multiple logistic regression are fulfilled. Adjusted odds ratios (AOR) with 95% confidence

intervals (CI) were used to measure strength of the association between outcome variables (STH) and its associated factors. Finally, p-value <0.05 declared a significant association.

Operational definitions

Soil-transmitted helminth (STH) infections were identified in children through microscopic detection of *A. lumbricoides, T. trichiura,* hookworms, schistosomiasis, or other helminths.

Intensity of *A. lumbricoides* Light Infection (1–4,999 egg per gram), Moderate infection (5,000 – 49,999 egg per gram) and (>50,000 egg per gram) (26).

Intensity of *T. trichiura* Light Infection (1–999 eggs per gram), Moderate infection (1,000–9,999 eggs per gram), and Heavy infection (>10,000 eggs per gram) (26).

Intensity of Hookworms Light Infection (1–1,999 eggs per gram), Moderate infection (2,000–3,999 eggs per gram) and Heavy infection (>4,000 eggs per gram) (26).

Ethical Approval

Ethical clearance was obtained from Addis Ababa University Akililu Lemma Institute of Health Research Institutional Research Ethics Committee. Permission was obtained from sub city health and education department to each selected school. All participants informed well about the purpose and the procedures of the study. All responses were kept confidential and anonymous. Participation was fully voluntary and written informed consent was obtained from each participant.

Results

Socio-demographic characteristics

A total of 394 participants were interviewed yielding an overall response rate of 94%.

The mean age of participants' school children in years was 11.7 with a standard deviation of ±2.3 years and 252 (64.0%) were aged ≤12 years, and 212 (53.8%) were female. Most children 383 (97.2%) lived in rural areas. Regarding the educational status of the mother, 205 (52.0%) had primary education, while 50 (12.7%) had no formal education, and 30 (7.6%) had completed college or above. For fathers, 23 (5.8%) had no formal education, and 77 (19.5%) had completed college or above. Concerning the children's educational level, 171 (43.4%) were in grades 5-6, while 112 (28.4%) were in grades 1-4. In terms of occupation, 315 (79.9%) of mothers were housewives, and 120 (30.5%) of fathers worked in private business. Most children 387 (98.2%) lived with their parents, and 238 (60.4%) came from households categorized as poor in wealth status. Regarding family size, 268 (68.0%) of the children came from families with ≤5 members, and 335 (85.0%) had fathers who did not practice polygamy (Table 1).

Table 1: Socio -demographic and socio-economic characteristics of schoolchildren in Tula Sub-city of Hawassa city, Sidama, Ethiopia in 2025.

**Variables**		**Frequency**	**Percent**
Age group in year	≤ 12	252	64.0
	> 12	142	36.0
Gender	Male	182	46.2
	Female	212	53.8
Place of residence	Urban	11	2.8
	Rural	383	97.2
Mother's Education	No formal education	50	12.7
	Primary	205	52.0
	Secondary	109	27.7
	College and above	30	7.6
Father's Education	No formal education	23	5.8
	Primary	128	32.5
	Secondary	166	42.1
	College and above	77	19.5
Highest Education Level of Child (Grade)	1-4	112	28.4
	5-6	171	43.4
	6-8	111	28.2
Mother's Occupation	Housewife	315	79.9
	Gov't employee	37	9.4
	Private business	32	8.1
	Student	2	0.5
	Others	8	2.0
Father's Occupation	Farmer	131	33.2
	Student	7	1.8
	Private business	120	30.5
	Daily Laborer	37	9.4
	Gov't employee	97	24.6
	Others	2	0.5
Husband had polygamy	No	335	85.0
	Yes	59	15.0
Child's Living Condition	With parents	387	98.2
	Relative	7	1.8

WASH and Other related characteristics

Out of 394 participants, regarding WASH and related characteristics, 315 (79.9%) of households had pipe water, 18.0% used public taps, and 2.0% relied on well water or hand pumps. At school level, 51.8% of children had no water access, while 48.2% had pipe water. In terms of water treatment, 64.0% of households did not treat water before drinking, and 95.9% did not store drinking water separately. The water usage, 66.2% used one 20L jerry can, and 30.5% used two. Half of the participants washed hands after soil contact, and 32.5% washed fruits and vegetables before eating. Additionally, 77.4% of children swam or bathed in streams, or lakes (Supplementary table 1).

Regarding health-related characteristics, 276 (70.1%) of schoolchildren experienced health-related illnesses in the past two weeks. The majority, 216 (54.8%), had constipation or diarrhea, while 177 (44.9%) had a cough. A significant number, 252 (64.0%), experienced abdominal pain, and 245 (62.2%) had loss of appetite. Most schoolchildren 393 (99.7%) did not walk barefoot or use anti-helminthic drugs. Regarding delivery, 375 (95.2%) were delivered vaginally and only 174 (44.2%) received deworming treatment (Table 2).

The Distribution of Soil-Transmitted Helminths

The overall prevalence of STH was ~~found to be~~ 42.6% (168/??) (95% CI [(37.8-47.6%]. Among the STHs, *A. lumbricoides* was the most prevalent (29.2%) [95% CI [24.9-33.8%], -followed by *T. trichiura* with (17.8%) 95%CI [(14.2-21.8%], *hookworms*. (5.1%) 95% CI [(3.2-7.6%], and *Schistosoma* mansoni (3.3%13/???) 95% CI [(1.9-5.4%)] (Figure 1).

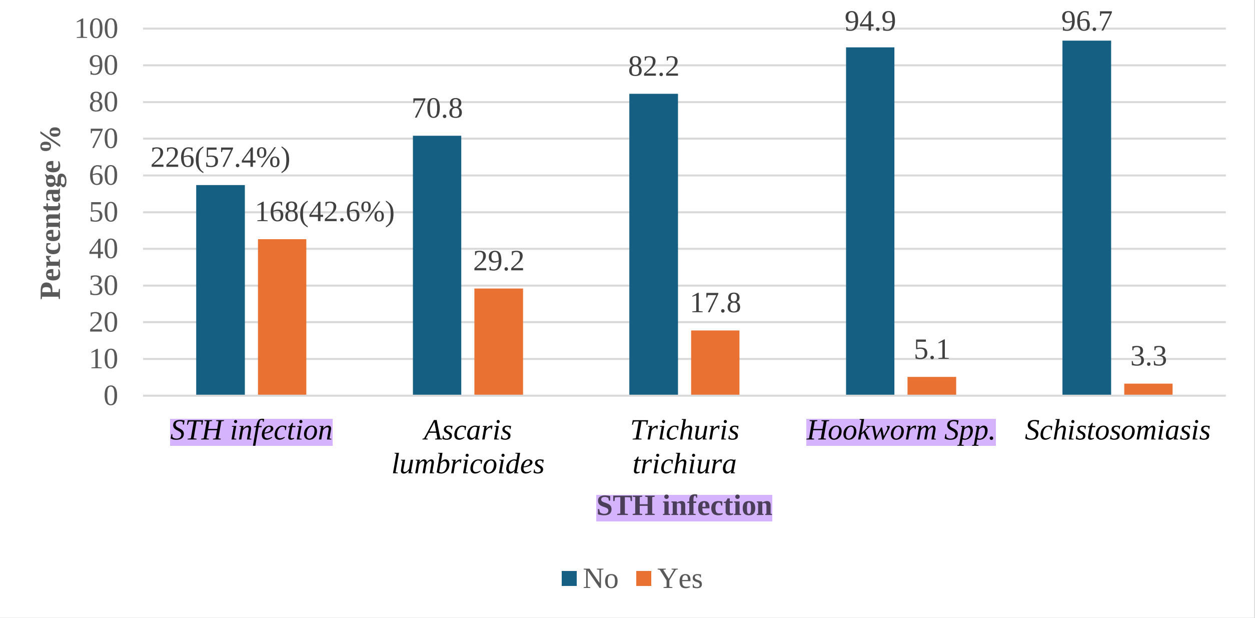

Figure 1: Prevalence of soil-transmitted helminths among schoolchildren in Hawella Tula Rural district, Hawassa, Sidama Regional State, Ethiopia 2025.

Factors Associated with soil-transmitted helminthiasis among schoolchildren

In bivariate logistic regression, socio-demographic, economic, environmental, behavioral, and WASH-related factors with $p < 0.25$ were selected for multivariable analysis. The multivariable model identified significant predictors of *A. lumbricoides* and *T. trichiura* infections in schoolchildren at $p < 0.05$. In multivariate logistic regression, STH / Shistosomia risk was higher among children who ate unwashed fruits/vegetables (AOR 2.89, 95% CI: 1.73–4.85), swam in streams/lakes (AOR 2.23, 95% CI: 1.31–3.79), lacked deworming treatment (AOR 2.12, 95% CI: 1.26–3.56), as compared with their counter parts (Table 2).

Table 2: Multivariate logistic regression associated with soil-transmitted helminthes and shistosomes among schoolchildren in Hawella Tula Rural district, Hawassa, Sidama Regional State, Ethiopia, 2025.

	**STH infection**						
	**Yes**		**No**				
	**Freq.**	**%**	**Freq.**	**%**	**COR (95% Cl)**	**AOR (95% Cl)**	**P-Value**
Maternal education							
No formal education	19	38.0	31	62.0	1	1	
Primary education	96	46.8	109	53.2	1.44 (0.76, 2.71)	1.46 (0.72, 2.95)	0.298
Secondary & above	53	38.1	86	61.9	1.01 (0.52, 1.96)	1.09 (0.52, 2.31)	0.815
Washing fruits/vegetables before eating							
No	136	51.1	130	48.9	3.14 (1.97, 5.01)	2.89 (1.73, 4.85)	<0.001*
Yes	32	25.0	96	75.0	1	1	
Habit of swimming or bathing in streams/lake							
Yes	48	53.9	41	46.1	1.81 (1.12, 2.91)	2.23 (1.31, 3.79)	0.003*
No	120	39.3	185	60.7	1	1	
Had deworming treatment							
No	137	48.4	146	51.6	2.42 (1.51, 3.90)	2.12 (1.26, 3.56)	0.004*
Yes	31	27.9	80	72.1	1	1	
Water treatment before drinking							
No	121	48.0	131	52.0	1.87 (1.22, 2.86)	1.60 (1.00, 2.55)	0.051
Yes	47	33.1	95	66.9	1	1	

NB: * statistically significant on multivariate analysis p-value (<0.05), **COR:** crude odds ratio, **AOR:** adjusted odds ratio, **CI:** confidence interval, **P-V**: p -value, **1:** reference

Schoolchildren were more likely to be exposed to A. *lumbricoides*; who ate unwashed fruits/vegetables (COR 2.24, 95% CI: 1.26–3.98), lacked deworming treatment (AOR 2.34, 95% CI: 1.30–4.23), as compared with their counter parts (supplementary table-2).

Furthermore, a child who had no deworming treatment had a AOR of 6.64 (95% CI: 1.92, 11.21) were more likely to be exposed to *T. trichiura* among schoolchildren as compared with their counter parts (supplementary table-3).

Discussions

The present cross-sectional study showed that the overall prevalence of soil-transmitted helminths among the school children in Hawella Tula Rural district, in Hawassa was 42.6%. Among the identified STHs, *A. lumbricoides* was the most prevalent (29.2%) followed by*T. trichiura* (17.8%) and h*ookworms* (5.1%), and schistosomiasis (3.3%). The present findings of STH prevalence was consistent with previous studies who reported 38.2% from southwest Nigeria (27), 46.7% from Jimma, Oromia, Ethiopia (28) and 39.0% from Northwest Ethiopia (29). However, the current finding was lower as compared to 54% Yirgachefee, South Ethiopia (13), 84.4% in Mettu town, Southwest Ethiopia (14), 50.0% in Kola Diba Primary School, Northwest Ethiopia (30) and 67.7% in Gara Riketa primary school children at Hawassa Tula Sub-City, Southern Ethiopia (15). On the contrary, the findings of the present study was higher as compared to 31.2% in North-West Ethiopia (16), 27.1% in Birbir town, Southern Ethiopia (31) and 36.1% in Rwanda (32). The above observed discrepancies in soil-transmitted helminth prevalence can be attributed to differences in geographical conditions, diagnostic methods, and variations in hygiene practices, deworming programs, and health interventions. Additionally, climatic factors, urbanization levels, and

behavioral differences, such as barefoot walking and sanitation habits could potentially contribute to the variation in prevalence of STH (33).

The current study showed that that children who used to eat unwashed fruits or vegetables before were more likely to be exposed to soil-transmitted helminths (*A. lumbricoides*). The current finding was consistent and in agreement with that reported from Birbir town in southern Ethiopia (15), southern Ethiopia (30) and in rural western Uganda (34). The observed scenario can be associated with the habit of children who eat unwashed fruits or vegetables and creating an opportunity for exposure to STH due to contamination with helminth eggs from soil, human feces, or unclean water, which facilitates transmission (35).

This study revealed that schoolchildren who swam or bathed in streams or lakes faced a higher risk of STH infection. Similar findings were also reported from southern Ethiopia (27) and north-west Ethiopia (16). Some of the possible and likely reasons and factors could be the fact that schoolchildren can easily be exposed to contaminated water sources, which may contain helminthes eggs or larvae from human and animal feces (35).

On the other hand, the findings of the current study indicated that children who had no deworming treatment were more likely to be exposed to STH including *A. lumbricoides* and *Trichuris trichiura*. The same results and observations were recoded from Yirgacheffee, South Ethiopia (13), Southwest Ethiopia (14), North-West Ethiopia (16) and Southern Ethiopia (15). Absence of deworming practices might be linked to continuous cycle of infection, lack of parasite elimination, and increased risk of reinfection from contaminated environments (36).

In nutshell, the overall high prevalence of STH highlights the significant impact of hygiene practices, access to clean water, deworming programs, and food security on the risk of soil-transmitted helminthes infections among schoolchildren. Poor sanitation, contaminated water sources, and lack of deworming, contribute to increased vulnerability, leading to persistent infections and negative health outcomes (37).

Limitations

There might be a potential for recall and social desirability bias in the child feeding practice and socioeconomic. In addition, since the study is cross-sectional, it may not be strong to demonstrate direct cause and effect relationship between risk factors and outcome. Since the study included children, there is also a potential for recall bias among respondents answering questions relating to events happening in the past two years and above; taking extra meal during pregnancy or lactation, & dietary diversity of children in the past 24 hrs.

Conclusions

The present study showed that more than one-third of the schoolchildren in the study area were affected by STH and schistosomiasis, indicating a convincing need for enhanced interventions. Several factors including eating unwashed fruits and vegetables, exposure to contaminated water from swimming or bathing in streams or lakes, lack of deworming treatment, and severe food insecurity were found to increase the risk of STH infections. Stunted growth was also associated with higher susceptibility to STH. Furthermore, nearly one-third of schoolchildren in the study area are stunted or underweight. The findings emphasize the importance of improving hygiene,

expanding deworming programs, and addressing food insecurity to mitigate the spread of these infections among children.

Abbreviations

AOR= Adjusted Odds Ratio, COR= Crudes Odds Ratio, DDS= Dietary Diversity Score, EDHS=Ethiopian Demographic and Health Survey, ETB= Ethiopian birr, SPSS= Statistical Package for Social Science, UNICEF: United Nations International Children's Emergency Fund, WHO= World Health Organization

Declarations

Consent for publication

Not applicable.

Availability of data and materials

There is no remaining pertinent data and materials, all information is presented in the main manuscript. The questionnaire and a dataset used in this study are available from the corresponding author on reasonable request.

Competing interests

The authors declare that they have no conflict of interests.

Funding

Student research support fund provided for the study from Addis Ababa and Hawassa University. The funder had no role in designing the study and conducting the analysis.

Authors' contributions

FB[1*], MM[1] BE HA [2] BT[3] wrote the conception, study design, execution, acquisition of data, analysis and interpretation, or in all these areas and read and approved the final manuscript. All authors contributed to data analysis, drafting or revising the article, have agreed on the journal to which the article will be submitted, and gave final approval of the version to be published.

Acknowledgments

The authors would like to thank Addis Ababa University, Colgate University, USA and Hawassa University for financial, and material support and laboratory setup for the study. The authors are also grateful to all data collectors and study participants for their valuable contributions.

References

1. Montresor A, Mupfasoni D, Mikhailov A, Mwinzi P, Lucianez A, Jamsheed M, *et al.* The global progress of soil-transmitted helminthiases control in 2020 and World Health Organization targets for 2030. PLoS neglected tropical diseases. 2020;14(8):e0008505.

2. Gbonhinbor J, Abah A E, Awi-Waadu G. Prevalence of intestinal parasitic infection and associated risk factors among primary school-aged children (5-15 years) in Southern Nigeria. Int J Infect. 2022;9(3):e123721.

3. Amoadu M, Abraham S A, Adams A K, Akoto-Buabeng William, Obeng Paul, Hagan Jr John Elvis. Risk factors of malnutrition among in-school children and adolescents in developing countries: A scoping review. Children. 2024;11(4):476.

4. Seyoum D., Tsegaye R., Tesfaye A. Under nutrition as a predictor of poor academic performance; the case of Nekemte primary schools students, Western Ethiopia. BMC Res Notes. 2019;12(1):727.

5. Morales F, Montserrat-De la Paz S, Leon M J, Rivero-P F. Effects of malnutrition on the immune system and infection and the role of nutritional strategies regarding improvements in children's health status: A literature review. Nutrients. 2023;16(1):1.

6. Organization World Health. 2030 targets for soil-transmitted helminthiases control programmes: World Health Organization; 2020.

7. Organization World Health. Global report on neglected tropical diseases 2023: World Health Organization; 2023.

8. Control Centers for Disease, Prevention. CDC-soil-transmitted helminths. Centers for Disease Control and Prevention Retrieved November. 2020;17:2021.

9. Organization World Health. Working to overcome the global impact of neglected tropical diseases: first WHO report on neglected tropical diseases: World Health Organization; 2010.

10. Organization WHO-World Health. Schistosomiasis and soil-transmitted helminthiases number of people treated in 2018. Wkly Epidemiol Rec. 2019;50(94):601-12.

11. FDRE (Federal Democratic Republic of Ethiopia). National Nutrition Program, 2016-2020. 2016.

12. UNICEF. Reducing Stunting In Children Under 5 Years Of Age: A Comprehensive Evaluation of Unicef's Strategies and Programme Performance Global Synthesis Report. New York: UNICEF; 2017.

13. Molla E., Mamo H. Soil-transmitted helminth infections, anemia and undernutrition among schoolchildren in Yirgacheffee, South Ethiopia. BMC Res Notes. 2018;11(1):585.

14. Yeshanew S., Bekana T., Truneh Z., Tadege M., Abich E., Dessie H. Soil-transmitted helminthiasis and undernutrition among schoolchildren in Mettu town, Southwest Ethiopia. Sci Rep. 2022;12(1):3614.

15. Eyamo T., Girma M., Alemayehu T., Bedewi Z. Soil-Transmitted Helminths And Other Intestinal Parasites Among Schoolchildren In Southern Ethiopia. Res Rep Trop Med. 2019;10:137-43.

16. Feleke D. G., Ali A., Bisetegn H., Debash H., Birara W., Andualem A. Soil-transmitted helminthes and Schistosoma mansoni infections among primary school children at Ambasame primary school, North-West Ethiopia: a cross-sectional study. BMC Pediatr. 2022;22(1):477.

17. Aemiro A., Menkir S., Tegen D., Tola G. Prevalence of Soil-Transmitted Helminthes and Associated Risk Factors Among People of Ethiopia: A Systematic Review and Meta-Analysis. Infect Dis (Auckl). 2022;15:11786337211055437.

18. Health Federal Democratic Republic Of Ethiopia Ministry of. Ethiopian Demography and Health Survey 2016.

19. Workneh T, Esmael A, Ayichiluhm M. Prevalence of intestinal parasitic infections and associated factors among Debre Elias primary schools children, East Gojjam Zone, Amhara Region, North West Ethiopia. J Bacteriol Parasitol. 2014;5(1):1.

20. Godana W, Mengistie B. Exploring barriers related to the use of latrine and health impacts in rural Kebeles of Dirashe district Southern Ethiopia: implications for community lead total sanitations. Health Science Journal. 2017;11(2):1.

21. Negussu N., Mengistu B., Kebede B., Deribe K., Ejigu E., Tadesse G., *et al.* Ethiopia Schistosomiasis and Soil-Transmitted Helminthes Control Programme: Progress and Prospects. Ethiop Med J. 2017;55(Suppl 1):75-80.

22.Tafese B, Christine L , Simon R , et al. Biesalski Validation of the CIMI-Ethiopia Program and Seasonal Variation in Maternal Nutrient Intake in Enset (False Banana) Growing Areas of Southern Ethiopia. Int. J. Environ. Res. Public Health 2019, 16, 2852; doi:10.3390/ijerph16162852

23. World Health Organization. Bench aids for the diagnosis of intestinal parasites, 2nd ed Geneva: World Health Organization; 2019.

24. Ranganathan P, Deo V, Pramesh CS. Sample size calculation in clinical research. Perspect Clin Res. 2024 Jul-Sep;15(3):155-159. doi: 10.4103/picr.picr_100_24. Epub 2024 Jul 4. PMID: 39140012; PMCID: PMC11318789.

25. DeWorm3 Standard Operating Procedure 707 : Kato-Katz Preparation and Analysis

26. World Health Organization. 749. Geneva, Switzerland: World Health Organization; 1987. Prevention and control of intestinal parasitic infections.

27. Adenusi A A., Sheba K F., Ugwueze K T., Akinsola O J., Adegbite R B., Nwanya V A., *et al.* Community-based prevalence, intensity and risk factors associated with soil-transmitted helminthiases and intestinal schistosomiasis in Apojola, Ogun state, southwest Nigeria. BMC Infectious Diseases. 2024;24(1):1302.

28. Abdissa D., Kebede Y., Sudhakar M., Abraham G., Bulcha G., Shiferaw T., *et al.* Communities' knowledge, perceptions and preventive practices on soil-transmitted helminthes in Jimma, Oromia, Ethiopia: Formative mixed study. PLoS Negl Trop Dis. 2024;18(9):e0012483.

29. Muluneh C., Hailu T., Alemu G. Prevalence and Associated Factors of Soil-Transmitted Helminth Infections among Children Living with and without Open Defecation Practices in Northwest Ethiopia: A Comparative Cross-Sectional Study. Am J Trop Med Hyg. 2020;103(1):266-72.

30. Hussein A, Alemu M, Ayehu A. Soil Contamination and Infection of School Children by Soil-Transmitted Helminths and Associated Factors at Kola Diba Primary School, Northwest Ethiopia: An Institution-Based Cross-Sectional Study. Journal of Tropical Medicine. 2022;2022(1):4561561.

31. Alemu G, Abossie A, Yohannes Z. Current status of intestinal parasitic infections and associated factors among primary school children in Birbir town, Southern Ethiopia. BMC Infectious Diseases. 2019;19(1):270.

32. Irisarri-Gutierrez M. J., Acosta L., Parker L. A., Toledo R., Bornay-Llinares F. J., Esteban J. G., *et al.* Anemia and undernutrition in intestinally parasitized schoolchildren from Gakenke district, Northern Province of Rwanda. PLoS One. 2022;17(1):e0262361.

33. Li, X.; Stringer, L.C.; Dallimer, M. The Impacts of Urbanisation and Climate Change on the Urban Thermal Environment in Africa. *Climate* **2022**, *10*, 164.

34. Ojja S, Kisaka S, Ediau M, Tuhebwe D, Kisakye A N, Halage A A, *et al.* Prevalence, intensity and factors associated with soil-transmitted helminths infections among preschool-age children in Hoima district, rural western Uganda. BMC infectious diseases. 2018;18:1-12.

35 .Gurmassa, B.k., Gari, S.R., Solomon, E.T. *et al.* Distribution of helminth eggs in environmental and stool samples of farming households along Akaki River in Addis Ababa, Ethiopia. *Trop Med Health* **51**, 67 (2023).

36.Nachaiwieng, W., Sanit, S., Kongta, N. *et al.* The impact of an integrated intervention program combining drug therapy with water, sanitation, and hygiene (WASH) education on reinfection with intestinal parasitic infections among the Karen hill tribe in northern Thailand. *Parasites & Vectors* **17**, 544 (2024).

37. Susana V N, Amy J. P, Ebba A, *et al.* The role of water, sanitation and hygiene interventions in reducing soil-transmitted helminths: interpreting the evidence and identifying next steps. *Parasites & Vectors (2019) 12:273*

Supplementary Table 1: WASH and Other related characteristics of schoolchildren in southern Ethiopia, 2025.

Variables	No.	(%)

Water Sources (HH)		
Pipe water	315	(79.9)
Public tap	71	(18.0)
Well water, and hand pump	8	(2.0)
Child's Water at School		
None	204	(51.8)
Pipe water	190	(48.2)
Water Treatment Before Drinking		
No	252	(64.0)
Yes	142	(36.0)
Separate Storage of Drinking Water		
No	378	(95.9)
Yes	16	(4.1)
Water Use (20L Jerry Can)		
1	261	(66.2)
2	120	(30.5)
≥ 3	13	(3.3)
Handwashing After Soil Contact		
No	194	(49.2)
Yes	200	(50.8)
Washing Fruits/Vegetables Before Eating		
No	266	(67.5)
Yes	128	(32.5)
Avoid Swimming or Bathing in Rivers/Ponds/Streams/Lakes		
No	89	(22.6)
Yes	305	(77.4)

supplementary Table- 2: Multivariate logistic regression associated with Ascaris *lumbricoides* among schoolchildren in southern Ethiopia, 2025.

	Ascaris *lumbricoides*						
	Positive		Negative				
	Freq.	(%)	Freq.	(%)	COR (95% Cl)	AOR (95% Cl)	P-Value
Residence							
Urban	6	(54.5)	5	(45.5)	1	1	
Rural	109	(28.5)	274	(71.5)	3.02 (0.90, 10.09)	2.35 (0.66, 8.43)	0.190
Age of Child							
≤ 12	64	(25.4)	188	(74.6)	1	1	
> 12	51	(35.9)	91	(64.1)	1.65 (1.06, 2.57)	1.36 (0.82, 2.25)	0.237
Washing Fruits/Vegetables Before Eating							
No	94	(35.3)	172	(64.7)	2.79 (1.64, 4.74)	2.24 (1.26, 3.98)	0.006*
Yes	21	(16.4)	107	(83.6)	1	1	
Had deworming treatment							
No	96	(33.9)	187	(66.1)	2.49 (1.43, 4.32)	2.34 (1.30, 4.23)	0.005
Yes	19	(17.1)	92	(82.9)	1	1	
Water Treatment Before Drinking							
No	84	(33.3)	168	(66.7)	1.79 (1.11, 2.88)	1.58 (0.95, 2.63)	0.081
Yes	31	(21.8)	111	(78.2)	1	1	
Household food insecurity							
Food secure	19	(21.1)	71	(78.9)	1	1	
Mild/Moderately insecure	76	(29.9)	178	(70.1)	1.60 (0.90, 2.83)	1.54 (0.83, 2.85)	0.171
Severely insecure	20	(40.0)	30	(60.0)	2.49 (1.17, 5.32)	2.90 (1.24, 6.77)	0.014*
Height for Age							
Normal height	67	(24.7)	204	(75.3)	1	1	
Stunted	48	(39.0)	75	(61.0)	1.95 (1.24, 3.07)	1.72 (1.01, 2.93)	0.047*
BMI for Age							
Normal weight	70	(26.7)	192	(73.3)	1	1	
Under weight	45	(34.1)	87	(65.9)	1.42 (0.90, 2.23)	1.26 (0.72, 2.21)	0.418

supplementary Table-3: Multivariate logistic regression associated with Ascaris *lumbricoides* among schoolchildren in southern Ethiopia, 2025.

	**Trichuris *trichiura***						
	**Positive**		**Negative**				
	**Freq.**	**(%)**	**Freq.**	**(%)**	**COR (95% Cl)**	**AOR (95% Cl)**	**P-Value**
Washing Fruits/Vegetables Before Eating							
No	56	(21.1)	210	(78.9)	2.17 (1.16, 4.07)	1.73 (0.89, 3.37)	0.109
Yes	14	(10.9)	114	(89.1)	1	1	
Avoid swimming or bathing in streams/lake							
No	21	(23.6)	68	(76.4)	1.61 (0.91, 2.87)	1.70 (0.93, 3.12)	0.086
Yes	49	(16.1)	256	(83.9)	1	1	
Had deworming treatment							
No	64	(22.6)	219	(77.4)	5.11 (2.15, 12.19)	4.64 (1.92, 11.21)	0.001*
Yes	6	(5.4)	105	(94.6)	1	1	
Water treatment before drinking							
No	49	(19.4)	203	(80.6)	1.39 (0.80, 2.43)	1.21 (0.67, 2.18)	0.536
Yes	21	(14.8)	121	(85.2)	1	1	
Dietary diversity score							
Good	14	(12.0)	103	(88.0)	1	1	
Poor	56	(20.2)	221	(79.8)	1.86 (0.99, 3.50)	1.90 (0.98, 3.68)	0.056

Supplementary Table 1: WASH and Other related characteristics of schoolchildren in southern Ethiopia, 2025.

**Variables**	**No.**	**(%)**
Water Sources (HH)		
Pipe water	315	(79.9)
Public tap	71	(18.0)
Well water, and hand pump	8	(2.0)
Child's Water at School		
None	204	(51.8)
Pipe water	190	(48.2)
Water Treatment Before Drinking		
No	252	(64.0)
Yes	142	(36.0)
Separate Storage of Drinking Water		
No	378	(95.9)
Yes	16	(4.1)
Water Use (20L Jerry Can)		
1	261	(66.2)
2	120	(30.5)
≥ 3	13	(3.3)
Handwashing After Soil Contact		
No	194	(49.2)
Yes	200	(50.8)
Washing Fruits/Vegetables Before Eating		
No	266	(67.5)
Yes	128	(32.5)
Avoid Swimming or Bathing in Rivers/Ponds/Streams/Lakes		
No	89	(22.6)

Yes	305	(77.4)

supplementary table- 2: Multivariate logistic regression associated with Ascaris *lumbricoides* among schoolchildren in southern Ethiopia, 2025.

	**Ascaris *lumbricoides***						
	**Positive**		**Negative**				
	**Freq.**	**(%)**	**Freq.**	**(%)**	**COR (95% Cl)**	**AOR (95% Cl)**	**P-Value**
Residence							
Urban	6	(54.5)	5	(45.5)	1	1	
Rural	109	(28.5)	274	(71.5)	3.02 (0.90, 10.09)	2.35 (0.66, 8.43)	0.190
Age of Child							
≤ 12	64	(25.4)	188	(74.6)	1	1	
> 12	51	(35.9)	91	(64.1)	1.65 (1.06, 2.57)	1.36 (0.82, 2.25)	0.237
Washing Fruits/Vegetables Before Eating							
No	94	(35.3)	172	(64.7)	2.79 (1.64, 4.74)	2.24 (1.26, 3.98)	0.006*
Yes	21	(16.4)	107	(83.6)	1	1	
Had deworming treatment							
No	96	(33.9)	187	(66.1)	2.49 (1.43, 4.32)	2.34 (1.30, 4.23)	0.005
Yes	19	(17.1)	92	(82.9)	1	1	
Water Treatment Before Drinking							
No	84	(33.3)	168	(66.7)	1.79 (1.11, 2.88)	1.58 (0.95, 2.63)	0.081
Yes	31	(21.8)	111	(78.2)	1	1	
Household food insecurity							
Food secure	19	(21.1)	71	(78.9)	1	1	
Mild/Moderately insecure	76	(29.9)	178	(70.1)	1.60 (0.90, 2.83)	1.54 (0.83, 2.85)	0.171
Severely insecure	20	(40.0)	30	(60.0)	2.49 (1.17, 5.32)	2.90 (1.24, 6.77)	0.014*
Height for Age							
Normal height	67	(24.7)	204	(75.3)	1	1	
Stunted	48	(39.0)	75	(61.0)	1.95 (1.24, 3.07)	1.72 (1.01, 2.93)	0.047*
BMI for Age							
Normal weight	70	(26.7)	192	(73.3)	1	1	

Under weight	45	(34.1)	87	(65.9)	1.42 (0.90, 2.23)	1.26 (0.72, 2.21)	0.418

supplementary table-3: Multivariate logistic regression associated with Ascaris *lumbricoides* among schoolchildren in southern Ethiopia, 2025.

	**Trichuris *trichiura***						
	**Positive**		**Negative**				
	**Freq.**	**(%)**	**Freq.**	**(%)**	**COR (95% Cl)**	**AOR (95% Cl)**	**P-Value**
Washing Fruits/Vegetables Before Eating							
No	56	(21.1)	210	(78.9)	2.17 (1.16, 4.07)	1.73 (0.89, 3.37)	0.109
Yes	14	(10.9)	114	(89.1)	1	1	
Avoid swimming or bathing in streams/lake							
No	21	(23.6)	68	(76.4)	1.61 (0.91, 2.87)	1.70 (0.93, 3.12)	0.086
Yes	49	(16.1)	256	(83.9)	1	1	
Had deworming treatment							
No	64	(22.6)	219	(77.4)	5.11 (2.15, 12.19)	4.64 (1.92, 11.21)	0.001*
Yes	6	(5.4)	105	(94.6)	1	1	
Water treatment before drinking							
No	49	(19.4)	203	(80.6)	1.39 (0.80, 2.43)	1.21 (0.67, 2.18)	0.536
Yes	21	(14.8)	121	(85.2)	1	1	
Dietary diversity score							
Good	14	(12.0)	103	(88.0)	1	1	
Poor	56	(20.2)	221	(79.8)	1.86 (0.99, 3.50)	1.90 (0.98, 3.68)	0.056

Author Summary

Soil-transmitted helminths (STH) remain a public health problem in endemic areas. Ethiopia is known to be endemic for STH. However, epidemiological data on these infections needed for an effective control program are scarce or inexistent. For deworming implementation, passive strategies consisting of deworming of young children during vaccination campaigns and systematic treatment of children consulting at hospital for helminth-like symptoms are being implemented, as well as distribution of albendazole during primary school health education program school children's mass treatment. In the present work, we provide current Prevalence and intensity of soil-transmitted helminths and associated factors among School children in Hawella Tula Rural district, Hawassa, Sidama Regional State. which is the rural sub city among the 8 sub-cities of Hawassa City Administration. We reported a high prevalence of STH infections of low to moderate intensity in the study areas, where school age children are more at-risk age group. Our findings highlight the need to implement the WHO recommendations for control of STH infections in the country, such as mass drug administration of anthelminthic twice a year with great attention to school aged children. Health Education and other preventive measures such as WASH should be taken in consideration.

Related Manuscript

አዲስ አበባ ዩኒቨርሲቲ
አክሊሉ ለማ ፓቶባዮሎጂ [illegible]
አዲስ አበባ ፣ ኢትዮጵያ
1176
Fax: 251-11-2755296

ADDIS ABABA UNIVERSITY
Aklilu Lemma Institute of Pathobiology (ALIPB)
Addis Ababa, ETHIOPIA
251-11-276-30-91/213-57-25
e-mail: aklilu.lemma@aau.edu.et

Aklilu Lemma Institute of Pathobiology Institutional Research Ethics Review Committee (ALIPB-IRERC)

Ethical Clearance Certificate

Ref. No.: ALIPB IRERC/91/2015/22
Date: Decemeber 15, 2022

Title: "Soil transmitted helminthiasis associated outcomes, nutritional status and its impact on Gut microbiome Composition and Diversity among Gereha rikata School Children in Southern Ethiopia"

PI: Fisseha Bonja

Recommendation of the ALIPB Institutional Research Ethics Review Committee

Dear Fisseha,

The ALIPB-IRERC has reviewed your above mentioned research proposal and noted its scientific merit. The IRERC has approved your study and would like to remind you to submit progress reports of the work every 6 months and the final report upon completion of the study. Furthermore, you are expected to notify the ALIPB-IRERC ahead of time any amendments or modifications to the protocol or premature suspension or termination of the study.

STATUS: APPROVED

Needs NRERB clearance: **Yes: __ No: ✓**

IRERC Chairperson: **Alemshay Teka, PhD**
Signature:

IRERC Secretary: **Esayas Aklilu, PhD**
Signature:

Approval

Name: **Professor Mengistu Legesse, Director**
Signature:
Date: 24/12/[illegible]

ADDIS ABABA UNIVERSITY
Aklilu Lemma Institute of Patho biology

Cc// IRERC office

Abstract

Background: Soil-transmitted helminthiasis (STH) is prevalent in tropical regions due to poor sanitation and limited healthcare access, resulting in overall well-being, developmental and cognitive impairments, particularly among children. This study aimed to assess the prevalence of STH and associated factors among schoolchildren in the Sidama Regional State of Ethiopia. The findings highlight the urgent need for enhanced improved sanitation, health education and effective deworming initiatives.

Method: School-based cross-sectional study was conducted among 394 primary schoolchildren from June 2024 to December 2024. Data were collected using an interviewer-administered questionnaire. Infection with the parasite was diagnosed using Kato–Katz microscopic examination. Bivariate and multivariable logistic regression was used to identify factors associated with the STH infection. Results: The overall prevalence of STH is 168 (42.6%) with a 95% CI (37.8-47.6%). The most prevalence parasite was *A. lumbricoides* (n=115; 29.2%), *T. trichiura* (n=70; 17.8%), Hookworm spp (n=20; 5.1%), and Schistosomes (n=13; 3.3%). Children who ate unwashed fruits/vegetables AOR of 2.89 (95% CI: 1.73–4.85), swam or bathed in streams/lakes AOR of 2.23 (95% CI: 1.31–3.79), lacked deworming treatment AOR of 2.12 (95% CI: 1.26–3.56), were exposed to STHs including (A. lumbricoides, and T. trichiura).

Conclusion: Over one-third of schoolchildren had STH and Schistosoma. Factors that predict STH and Schistosoma include poor hygiene, lack of deworming, contaminated water, and; this implies the need for better hygiene, health education and deworming programs.

***Keywords*:** Prevalence, Intensity of STH, Factors; School children, Southern, Ethiopia
