# Supplementary material for: Prevalence, infection intensity, and risk factors of soil-transmitted helminthiasis and intestinal schistosomiasis among schoolchildren in Southern Ethiopia
Source: PLoS Negl Trop Dis. 2026 Mar 18;20(3):e0014115. doi: 10.1371/journal.pntd.0014115 (PMC13016474; doi:10.1371/journal.pntd.0014115)
Supplement: Attachment — Submitted filename: PNTD-D-25-01634 Reviewer Evaluation.docx [file pntd.0014115.s004.docx]

**Reviewer’s Evaluations**

**Prevalence and intensity of soil-transmitted helminthiasis and associated factors among school children in Hawella Tula Rural district, Hawassa, Sidama Regional State, Ethiopia**

| Journal: | *PLOS Neglected Tropical Diseases* |
| --- | --- |
| URL: | https://mc.manuscriptcentral.com/oryx |
| Manuscript Number: | PNTD-D-25-01634 |
| Keywords: | Prevalence, Intensity of STH, Schistosoma, Factors; School children,  Southern, Ethiopia |

**Reviewer’s Evaluation 1**

**Evaluation Report**

Kindly enter comments per section of the manuscript **(All comments should be detailed to provide adequate information for authors)**

| General comment | The manuscript presents a timely and relevant investigation into soil-transmitted helminths and schistosomiasis among schoolchildren. The study design, data presentation, and discussion are commendable. However, the entire work would benefit from substantial English language and structural improvement. Additionally, some sections contain redundancy and inconsistent formatting (spacing and punctuation). The authors should also clearly distinguish between STHs and schistosomiasis, as they are biologically and epidemiologically distinct infections. The manuscript would make a valuable contribution to parasitology research. |
| --- | --- |
| Abstract | - Line 13-15: Consider adding a sentence indicating that stool samples were collected for the detection of *Ascaris lumbricoides*, *Trichuris trichiura*, hookworms, and other parasites. - Line 18-20: Please separate STH (group the three intestinal worms together) from schistosomiasis (*S. mansoni*) in your text, reporting the parasite separately, and set *S. mansoni* apart. - Line 19-20: The Spelling is *Schistosoma mansoni,* or write it as intestinal schistosomiasis. - Line 23-26: Please revise punctuation and structure for clarity, replace the semicolon after “and” with a full stop, and consider splitting the sentence into two. - ***Keywords*:** I am suggesting Ethiopia, factors, intensity, prevalence, schoolchildren, *Schistosoma mansoni,* STH |
| Introduction | - Line 31-33: You can rewrite as Soil-transmitted helminthiasis (STH), caused by intestinal parasites such as *Ascaris lumbricoides*, *Trichuris trichiura*, and hookworms, remains a major public health concern in areas with poor sanitation and limited access to clean water. - Line 52-55: Please check the sentences again, prevalence was repeated twice. - The introduction addresses the prevalence of soil-transmitted helminthiasis (STH) at global, regional, and national levels. However, it does not adequately cover the other key components of the title, *intensity* and *associated factors*. The introduction should briefly emphasize the importance of infection intensity in assessing morbidity and provide background on common associated factors such as hygiene, sanitation, water source, and deworming practices. |
| Methodology | - Line 133 to 141: What about the intensity of *S. mansoni*? - Line 107: Please add if the qualitative responses were coded thematically. |
| Results | - Line 166: Your first mention of WASH should be written in full. - Line 186: Please complete the *S. mansoni* figure and write the term in Italics. - Line 187: I am suggesting that Figure 1 should read “Figure 1: Prevalence of soil-transmitted helminths and schistosomiasis among schoolchildren in Hawella Tula Rural district, Hawassa, Sidama Regional State, Ethiopia 2025. - What about the results/figure/table for the data on intensity? |
| Discussion | - The discussion is rich and well-structured. However, it can be revised. - Line 210-212: You might want to separate schistosomiasis from the STHs in phrasing since it’s not an STH, to avoid conceptual overlap. - Line 213-219: Please summarize. - Line 220-224: Please avoid repetition - Line 232-236: Consider distinguishing this as an *indirect* route, since STHs are mainly transmitted via soil or ingestion, not through skin penetration like schistosomiasis. - Line 239: Recorded, not recoded - Line 243-247: Avoid “In nutshell”, you can please use a more formal academic phrase, such as In summary or overall. - Line 166: When you say, all species are data deficient, do you mean all - Please take note of typos “that”, “that” - Always separate schistosomiasis from STH results to avoid conceptual confusion |
| Bibliography/References | The reference section is extensive. |
| Others(Author Summary) | - The Author Summary provides relevant background and emphasizes key public health concerns; however, the writing should be improved . Please consider restructuring the summary into a clear narrative that covers the context of the research, main findings, and implications.   That can be followed by perspectives and recommendations. |
| Final Note | This article at this stage needs a lot of corrections before acceptance |

Please rate the following: (1 = Excellent) (2 = Good) (3 = Fair) (4 = poor)

| Originality | 3 |
| --- | --- |
| Contribution To the Field | 2 |
| Technical Quality | 3 |
| Clarity Of Presentation | 3 |
| Depth Of Research | 3 |

**Recommendations**

Kindly mark with an X

| Accept As It Is |  |
| --- | --- |
| Requires Minor Corrections |  |
| Requires Moderate Revision |  |
| Requires Major Revision | **X** |
| Not Suitable for Further Processing (Please be clear and specific) |  |

**Additional Comments**

Please add any additional comments (Including comments/suggestions regarding online supplementary materials) if any
