# Supplementary material for: Prevalence, infection intensity, and risk factors of soil-transmitted helminthiasis and intestinal schistosomiasis among schoolchildren in Southern Ethiopia
Source: PLoS Negl Trop Dis. 2026 Mar 18;20(3):e0014115. doi: 10.1371/journal.pntd.0014115 (PMC13016474; doi:10.1371/journal.pntd.0014115)
Supplement: Attachment — Submitted filename: Response to Reviewers n.docx [file pntd.0014115.s006.docx]

**Responses to Reviewers**

**Manuscript ID:** PNTD-D-25-01634

**Prevalence and intensity of soil-transmitted helminthiasis, and associated factors among school children in Hawella Tula Rural district, Hawassa, Sidama Regional State, Ethiopia.**

*we appreciate your comments and welcome feedback from both the Editors and Reviewers on* this manuscript and hope that the amendments made below do justice to the comments and improve the manuscript.

**Journal Requirements:**

1) Please ensure that the CRediT author contributions listed for every co-author are completed accurately and in full.
At this stage, the following Authors/Authors require contributions: Berhanu Erko, Musa Mohammed Ali, Bineyam Taye, and Hagos Ashenafi. Please ensure that the full contributions of each author are acknowledged in the "Add/Edit/Remove Authors" section of our submission form.
The list of CRediT author contributions may be found here: https://journals.plos.org/plosntds/s/authorship#loc-author-contributions

Response: We thank the Editor for this suggestion, and have now completed contributions of all co-author (page 1, lines 12-25) as suggested.

2) Please provide an Author Summary. This should appear in your manuscript between the Abstract (if applicable) and the Introduction, and should be 150-200 words long. The aim should be to make your findings accessible to a wide audience that includes both scientists and non-scientists. Sample summaries can be found on our website under Submission Guidelines:
<https://journals.plos.org/plosntds/s/submission-guidelines#loc-parts-of-a-submission>

Response: Thanks, we have now added an Author summary between abstract and background of the study sections (page 3, line54-67) as suggested.

3**) Please upload all main figures as separate Figure files** in .tif or .eps format. For more information about how to convert and format your figure files please see our guidelines: 
<https://journals.plos.org/plosntds/s/figures>

Response: Thanks for the valuable comment – we have now uploaded the main figures as separate Figure files as suggested.

4) We notice that your supplementary Tables are included in the manuscript file. Please remove them and upload them with the file type 'Supporting Information'. Please ensure that each Supporting Information file has a legend listed in the manuscript after the references list.

Response: Thank you; we have amended the tables in the MS file and changed as table 4 in the MS file.

5) In the online submission form, you indicated that **"Raw data used** for preparation of this manuscript can be obtained from corresponding author with resealable request.". All PLOS journals now require all data underlying the findings described in their manuscript to be freely available to other researchers, either
1. In a public repository
2**.** Within the manuscript itself
3. **Uploaded as supplementary information.**
This policy applies to all data except where public deposition would breach compliance with the protocol approved by your research ethics board. If your data cannot be made publicly available for ethical or legal reasons (e.g., public availability would compromise patient privacy), please explain your reasons by return email and your exemption request will be escalated to the editor for approval. Your exemption request will be handled independently and will not hold up the peer review process, but will need to be resolved should your manuscript be accepted for publication. One of the Editorial team will then be in touch if there are any issues.

Response: Thank you, we have added few sentences in the data availability section (Page 22, line 355-356) “The authors confirm that all data underlying the findings are fully available without restriction. All relevant data are within the paper and its Supporting Information files.” in the revised manuscript.

**Reviewers' Comments:**

Reviewer's Responses to Questions

**Key Review Criteria Required for Acceptance?**

As you describe the new analyses required for acceptance, please consider the following:


**Methods:**

-Are the objectives of the study clearly articulated with a clear testable hypothesis stated?

-Is the study design appropriate to address the stated objectives?

-Is the population clearly described and appropriate for the hypothesis being tested?

-Is the sample size sufficient to ensure adequate power to address the hypothesis being tested?

-Were correct statistical analysis used to support conclusions?

-Are there concerns about ethical or regulatory requirements being met?

Reviewer #1: The study objectives are generally clear, however, the cross-sectional design appears appropriate for assessing prevalence and associated factors. The study population, school-aged children in the selected district is relevant and appropriate for the objectives. Information on sample size determination is clearly provided. The statistical analyses used appear suitable for descriptive purposes. Finally, ethical considerations was described, including ethical approval, and adherence to institutional or national research ethics guidelines.

Response: We thank Reviewer #1 for their positive assessment of the study's methodology, including the study design, target population, sample size calculation, statistical analysis, and ethical considerations

Reviewer #2: The method is well applied, although the sum of the parasitic infections is a matter that needs to be reconsidered.

Response: We thank the reviewer for the valuable suggestion. We have amended the parasitic infection totals accordingly, the combined total for STH and schistosomiasis, as well as separate totals for STH and Schistosoma mansoni infections in the result section (page 14, Line 246-251) “The overall prevalence of STH and intestinal schistosomiasis was found to be 42.6% (168/394) (95% CI [(37.8-47.6%]. the prevalence of STH 162 (41.1%) was infected, with a 95% CI of (36.3-46.0%). and *Schistosomiasis* with 13 (3.3%) and a 95% CI of (1.9-5.4%). Among the STHs, *A. lumbricoides* was the most prevalent 115/394 (29.2%) [95% CI [24.9-33.8%], followed by *T. trichiura* with 70/394 (17.8%) 95%CI [ (14.2-21.8%], *hookworms* 20/394(5.1%) 95% CI [ (3.2-7.6%], and *Schistosoma* mansoni (13/394) (3.3%)95% CI [ (1.9-5.4%)]” in the revised manuscript.

Reviewer #3: - The study aims to assess the prevalence and intensity of STH and identify associated factors among schoolchildren, a topic relevant and important public health objective. However, there was no explicitly stated testable hypothesis.

Response We thank the reviewer for this insightful comment. We agree that a clearly stated hypothesis strengthens the study's framework. In response, we have now added a few sentences at the end of the introduction section to address the testable hypothesis (page 5, lines 93-106) in the revised manuscript, which reads: “Despite various efforts to reduce the burden of soil-transmitted helminth (STH) infections and schistosomiasis in Ethiopia, the prevalence of these infections has never fallen below the World Health Organization (WHO) threshold for low prevalence. The goal of this threshold is to reduce morbidity from STH infections in preschool-aged children (pre-SAC) and school-aged children (SAC) by lowering the prevalence of moderate- or heavy-intensity infections to less than 1% in SAC. This ongoing burden is driven by factors including rapid reinfection post-treatment, entrenched environmental exposure risks, and complex socio-demographic determinants. As a result, continuous monitoring of the socio-demographic, environmental, and lifestyle characteristics associated with an increased risk of STH infections is essential. Tailoring interventions and control strategies to meet the specific needs of each community is critical. This study provides evidence on the prevalence and distribution of STH infections and intestinal schistosomiasis among schoolchildren in southern Ethiopia. It highlights a significant public health challenge and aims to inform targeted interventions for clinicians, public health officials, and policymakers to reduce the burden of STH infections and improve child health.*”*

- The study adopted a cross-sectional design, which is appropriate for estimating prevalence and identifying associations; however, this design limits causal inference.

Response- We acknowledge this as one of the limitations, and have added a few sentences (page 20, line 320-323) in the discussion section to address this point, which reads now: “Our findings should be interpreted considering several limitations. We used a cross-sectional study design to collect baseline data on children's parasite infection status and predictors. This design limits our ability to draw causal inferences about the relationships between parasite status and identified risk factors.”

- The population is well described as schoolchildren (5–18 years) and their caregivers residing in Hawella Tula Rural district for at least six months. However, it is unclear if the choice of site was linked to disease burden, epidemiology, or other risk factors; this rationale should be clarified. Also, the abstract does not mention the study site. Line 106 notes that "data were collected using pre-tested, structured, and translated questionnaires"—please specify the language(s) used for translation.

Response - We are grateful to you for raising this point, and have added few sentences in the study population section (Page 6, Line126-128) “The study site, Hawella Tula Sub-City, was selected based on convenience, and all three primary schools within the area were included in the study.” in the revised manuscript.

- We are grateful to you for comments, and have added few sentences in the method section (page 6, line 109-110 ) “Hawella Tula sub city, Hawassa city, Sidama National Regional State, Ethiopia. (Figure 1 map of study area also included”.

- Thank you; we have amended the Questionnaires part (page 8, Line 155) read as “Data were collected through interviews using the Amharic -translated questionnaires.” in the revised manuscript.

- Sample size calculation is based on a prior prevalence estimate (54%) and includes a contingency margin. Although 420 participants were targeted, only 394 were analyzed. The impact of this shortfall on statistical power should be discussed. Questions to address include:
Response- We thank the reviewer for the valuable suggestion on Shortfall on statistical power, " Sample size was calculated using a prior prevalence of 54% with a target of 420 participants. Exclusions due to incomplete data (n=26), 394 participants were analysed. $n=394$the minimum detectable difference from 54% at 80% power and $\alpha=0.05$is ≈ 7.0 percentage points (compared with ≈ 6.8 points for $n=420$).”

i)**How many schools were sampled**?

Response: all three primary schools selected from Hawella Tula sub city

ii)**When and where were the samples analyzed**? On-site or in a laboratory?

Response: Thank you for highlighting this point. We established a temporary laboratory at each school compound, which enabled immediate microscopic examination without transporting samples elsewhere. We have updated this description in the Measurement and Data Collection section (page 8, line 162-165). “During the survey, stool samples were collected on-site and delivered to the laboratory within 10 minutes of defecation. A portion of each sample was examined immediately (within 10–30 minutes) to detect hookworm and other STH parasites, while the remaining portion was preserved for further analysis.” in the revised manuscript.

iii)**Within how many hours** of sample collection were analyses performed?

Response: “within10-30minuts after collection”

iv)**What criteria were used for school (site**) selection?

Response: “convenient sampling techniques used for sub city selection and all three primary schools within the area were included in the study”.

v)Were GPS coordinates collected for the schools? If yes, please provide a map showing their spatial distribution; if not, please clarify why.

Response: We thank the reviewer for this comment, “a Figure 1 map of study area also included and uploaded as separate Figure files (page 6, Line 110)” in the revised manuscript.

vi)How were participants classified as STH or S. mansoni positive, and how was worm intensity measured?

Response: Thank you for highlighting this point, we incorporating the suggested points to improve coherence and enhance clarity for readers (Page 9, Line171-175) “A child was classified as positive for soil-transmitted helminths (STH) if eggs of Ascaris lumbricoides, Trichuris trichiura, or hookworms (Ancylostoma duodenale or Necator americanus) were detected in the stool sample. Intestinal schistosomiasis was confirmed when Schistosoma mansoni eggs were identified. If no parasite eggs were observed in the processed slides, the child was classified as negative.” in the revised manuscript.

- Regarding intensity, we are incorporating the suggested points to improve coherence and enhance clarity for readers (page 9, Line 176-183)*.* “Infection intensity was classified according to WHO criteria. For Ascaris lumbricoides, light infection was defined as 1–4,999 eggs per gram (EPG), moderate as 5,000–49,999 EPG, and heavy as ≥50,000 EPG. For Trichuris trichiura, light, moderate, and heavy infections corresponded to 1–999 EPG, 1,000–9,999 EPG, and ≥10,000 EPG, respectively. Hookworm infection intensity was categorized as light (1–1,999 EPG), moderate (2,000–3,999 EPG), and heavy (≥4,000 EPG). For Schistosoma mansoni, infection was considered light at 1–99 EPG, moderate at 100–399 EPG, and heavy at ≥400 EPG” in the revised manuscript.” in the revised manuscript.

vii)What is the mass drug administration **(MDA) history of the health district**? Please provide the **number of treatment rounds delivered.**

Response: We thank the reviewer for this comment. “The district has conducted two rounds of MDA for STH and schistosomiasis within the last four years. Only Albendazole without praziquantel was provided to school-aged children during school-based campaigns."

- The use of bivariate and multivariable logistic regression is appropriate. Adjusted odds ratios (AORs) with 95% confidence intervals are reported. Although the Hosmer-Lemeshow test is mentioned, results are not presented and should be included to confirm model fit.

Response: We thank the reviewer for highlighting the need to report Hosmer-Lemeshow test results. We have now included these in the Results section (Page 9, Line 190-191): “The Hosmer-Lemeshow goodness-of-fit test indicated adequate model fit (χ²(8) = 6.34, p = 0.597).”

- Ethical clearance was obtained from Addis Ababa University Akililu Lemma Institute of Health Research Institutional Research Ethics Committee, and administrative permission was obtained from the sub-city health and education departments for each selected school. Informed consent procedures are clearly described, and responses were kept confidential and anonymous. Given participants included children as young as 5 years, it is unclear if assent was obtained and what the role of school teachers was in this process; this should be clarified.

Response: We are grateful to you for comments, and have added few sentences in the Ethical consideration section (page 11, Line 212-213) “written assent was obtained from each child and formal written consent was obtained from the parent/guardian.” in the revised manuscript.

**Results:**

-Does the analysis presented match the analysis plan?
-Are the results clearly and completely presented?
-Are the figures (Tables, Images) of sufficient quality for clarity?

Reviewer #1: The analysis presented does not fully align with the expected analysis plan, and some key results appear to be missing or insufficiently detailed. While the tables and figures included are generally relevant, they do not comprehensively capture all aspects of the study findings, particularly those related to infection intensity. The results section would benefit from clearer organization and complete reporting of all analyses outlined in the objectives.

Response: We thank the reviewer for this comment, and an infection intensity test results  and complete of all analysis included in the MS documents and figure uploaded as **s**eparate Figure files (page 14-15, Line 251-253) “Intensity of each parasite; Ascaris (moderate infection 54(13.7%) and Light infection 61(15.5%)), T.trichiura infection intensity heavy 2(0.5%), Moderate11(2.8%), Light 53(13.5%)) (Figure 3).”in the revised manuscript.

Reviewer #2: The study presents interesting results and corroborates the need for special attention to the health of schoolchildren for the effective elimination of STH. Furthermore, it also presents data on schistosomiasis, which is also a neglected disease waterborne disease whose elimination is between is among the WHO's Sustainable Development Goals. I suggest that the authors present the schistosomiasis data as additional data to the geohelminthiasis data, and not as a total sum.

Response: We thank the reviewer for this comment, and we recognize that and We have added a few sentences in the result sections will help the reader (page14, line 246-251) “The overall prevalence of STH and intestinal schistosomiasis was found to be 42.6% (168/394) (95% CI [(37.8-47.6%]. the prevalence of STH 162 (41.1%) was infected, with a 95% CI of (36.3-46.0%). and *Schistosomiasis* with 13 (3.3%) and a 95% CI of (1.9-5.4%). Among the STHs, *A. lumbricoides* was the most prevalent 115/394 (29.2%) [95% CI [24.9-33.8%], followed by *T. trichiura* with 70/394 (17.8%) 95%CI [ (14.2-21.8%], *hookworms* 20/394(5.1%) 95% CI [ (3.2-7.6%], and *Schistosoma* mansoni (13/394) (3.3%)95% CI [ (1.9-5.4%)] (Figure 2).” in the revised manuscript.

Reviewer #3: - The results follow the stated analysis plan by estimating prevalence, intensity classification, and identifying associated factors. However, intensity data including low, moderate, and high-intensity infection results for each of the STH species, including S. mansoni, is missing. Supplementary tables referenced, such as intensity data by site, are not included in the main document and should be appended.

Response: We thank the reviewer for this comment, and an infection intensity test results included in the MS document and figure uploaded in the separate Figure file (page 14-15, Line 251-253) “Intensity of each parasite; *A. lumbricoides* ( moderate infection 54(13.7%) and Light infection 61(15.5%)),*T.trichiura* infection intensity heavy 2(0.5%),Moderate11(2.8%), Light 53(13.5%))Hook worm infection intensity( Heavy 4(1%) Modereate 5(1.3%)Light11(2.8%)), Intestinal schistosomiasis intensity(heavy5(1.3%),Moderate 8(2%))(Figure 2)” in the revised manuscript.

- The study population is described as schoolchildren aged 5-18 and their caregivers, but worm prevalence data is not disaggregated by these two groups, as no denominator is provided. The results report the prevalence of S. mansoni, yet the study objectives do not include this. Please disaggregate the prevalence data by subgroup as appropriate.

Response: We revised the study population section. Since students’;” families or caregivers were not part of the study population but only responded to certain questions on behalf of the children, we added clarifying sentences and removed inappropriate text from the methodology section accordingly.(page 6,Line125-126) which now reads, “This school-based cross-sectional study focused on only primary schoolchildren (5–18 years) in Hawella Tula Rural district, Hawassa, Sidama Regional State, Ethiopia.*”* in the revised manuscript.

- Tables should be reformatted for improved clarity, with consistent use of column headers, proper alignment of percentages, and clearer separation of variable categories. It is unnecessary to include district, city, province, and country in the table labels. For example, Table 1 could be reworded from "Socio-demographic and socio-economic characteristics of schoolchildren in Tula Sub-city of Hawassa city, Sidama, Ethiopia in 2025" to simply "Socio-demographic and socio-economic characteristics of study participants." Figure 1 should specifically represent S. mansoni prevalence and not be labeled broadly as "schistosomiasis." Additionally, the label should capture S. mansoni prevalence rather than being generically titled "STH Infection."
-Response: Thank you, we have standardized the column headers, proper alignment of percentages, and clearer separation of variable categories in each table based on this recommendation in the revised manuscript

- Response: Thank you; we have amended the title of all the tables in the revised manuscript (page 13, line 228) “Table 1: Socio -demographic and socio-economic characteristics of study participants.”

-Response: Thank you; we have amended the title of the figure and unloaded in the separate figure file “Prevalence of soil-transmitted helminths and Intestinal **schistosomiasis** among study participants.” In the revised manuscript.

- Table 2 is captioned "Multivariate logistic regression associated with soil-transmitted helminths and **schistosomes** among schoolchildren in Hawella Tula Rural district, Hawassa, Sidama Regional State, Ethiopia, 2025," but no data regarding schistosomiasis is presented. This table is also distorted and difficult to interpret and should be reformatted for clarity.

Response: Thank you; we have amended the title of these table 2 by removing Schistosomes (page 15, Line263) in the revised manuscript.

- Line 207 references Supplementary Table 3, which is not provided in the document and should be included if referenced.

Response: Thank you; we have amended in the revised manuscript” as Table 4 in the document (page 18, line number 276.)”

**Conclusions:**

-Are the conclusions supported by the data presented?

-Are the limitations of analysis clearly described?

-Do the authors discuss how these data can be helpful to advance our understanding of the topic under study?

-Is public health relevance addressed?

Reviewer #1: The conclusions are generally consistent with the data presented, but they would be stronger if more explicitly tied to the key findings, particularly regarding infection intensity and associated risk factors. The discussion touches on public health implications, but it could better emphasize how the findings advance understanding of STH transmission dynamics and control strategies in endemic areas. The public health relevance is acknowledged but would benefit from clearer linkage to policy or intervention

Response: We thank the reviewer for their insightful comments on strengthening the conclusion and discussion. We agree that explicitly tying our conclusions to the key findings will enhance the manuscript's impact. In the revised version, we have: (page21, Line 329-345) “This study demonstrates that more than 40% of schoolchildren in Hawella Tula Rural District are infected with soil-transmitted helminths and *Schistosoma mansoni*, indicating a substantial and persistent burden in this endemic area. Key predictors of infection including consumption of unwashed fruits and vegetables, bathing in contaminated water sources, and lack of deworming highlight the important roles of environmental contamination, poor hygiene practices, and gaps in preventive interventions in sustaining transmission. These findings are consistent with previously published evidence and enhance our understanding of STH transmission dynamics by identifying behavioral and environmental factors that facilitate infection and reinfection among school-aged children.

Integrated control strategies, including regular school-based deworming, targeted health education to promote hygiene and safe food handling practices, and improvements in water, sanitation, and hygiene (WASH) infrastructure should be advocated as public health strategies. By linking infection patterns with actionable interventions; this study provides valuable evidence for policymakers and local health authorities to prioritize resources and implement context-specific programs aimed at reducing transmission, improving child health outcomes, and advancing national and global goals for the control of neglected tropical diseases. .”in the revised manuscript.

Reviewer #2: The conclusions are consistent and the study is relevant to public health.

Response: Thank you for comments!

Reviewer #3: - Conclusions are generally supported by the data, especially the associations between STH and hygiene behaviors, water exposure, and deworming. The need for enhanced intervention was cited but no examples provided.

Response: Thank you; we have amended in the revised manuscript (page21, line338-345) “Integrated control strategies, including regular school-based deworming, targeted health education to promote hygiene and safe food handling practices, and improvements in water, sanitation, and hygiene (WASH) infrastructure should be advocated as public health strategies. According to WHO guidelines, strength annual mass drug administration (MDA) is recommended in the study area. By linking infection patterns with actionable interventions; this study provides valuable evidence for policymakers and local health authorities to prioritize resources and implement context-specific programs aimed at reducing transmission, improving child health outcomes, and advancing national and global goals for the control of neglected tropical diseases.”

- Limitations could be expanded to include the low diagnostic sensitivity of Kato-Katz.

Response: Thank you; we have amended in limitation section (page20, Line 323-327) “low diagnostic sensitivity of the Kato-Katz (KK) technique is a widely recognized limitation, particularly when dealing with low sensitivity in Light Infections, Day-to-Day Variation, Time-Sensitive Processing, Sampling Effort and Requires Expertise. This limitation leads to the underestimation of infection prevalence in epidemiological surveys and can result in false-negative results.” In the revised manuscript.

- The paper doesn't inform if the WHO threshold for elimination as a public health problem (less than 2% of school-aged children have moderate or heavy intensity infections of any STH species) was achieved for STH. The paper fails to recommend if MDA is required and at what frequency, following WHO guidelines.

Response: Thank you; we have amended in conclusions section (page21, Line 340-342) “According to WHO guidelines, strength annual mass drug administration (MDA) is recommended in the study area.” in the revised manuscript.

**Editorial and Data Presentation Modifications?**

Use this section for editorial suggestions as well as relatively minor modifications of existing data that would enhance clarity. If the only modifications needed are minor and/or editorial, you may wish to recommend “Minor Revision” or “Accept”.

Reviewer #1: Major Revision

Reviewer #2: (No Response)

Reviewer #3: - Correct spelling errors such as “shistosomes” instead of “schistosomes”

Response: Thank you; we have corrected the spelling errors in the revised manuscript

- Ensure all denominators are included in prevalence figures.

Response: Thank you; we have amended in the revised manuscript result section (page 14, line 246-251) “The overall prevalence of STH and intestinal schistosomiasis was found to be 42.6% (168/394) (95% CI [(37.8-47.6%]. the prevalence of STH 41.1% (162/394) was infected, with a 95% CI of (36.3-46.0%). and Schistosomiasis with 3.3% (13/394) and a 95% CI of (1.9-5.4%). Among the STHs, *A. lumbricoides* was the most prevalent 115/394 (29.2%) [95% CI [24.9-33.8%], followed by *T. trichiura* with 70/394 (17.8%) 95%CI [ (14.2-21.8%], hookworms 20/394(5.1%) 95% CI [ (3.2-7.6%], and *Schistosoma* mansoni (13/394) (3.3%)95% CI [ (1.9-5.4%)]”

- Improve table formatting for clarity and consistency.

Response: Thank you; we have standardized the table format in the revised manuscript

- Include missing supplementary table.

Response: Thank you; we have amended in the revised manuscript” as Table 4 in the document (page 17-18, line number 275-276)”

- Standardize reference formatting and ensure completeness. For example, Word Health Organization (WHO) is written as "Organization World Health" in references 6,7,9 &10. UNICEF is abbreviated instead of written in full.

Response: Thank you; we have standardized the refences in the revised manuscript reference.

**Summary and General Comments**

Use this section to provide overall comments, discuss strengths/weaknesses of the study, novelty, significance, general execution and scholarship. You may also include additional comments for the author, including concerns about dual publication, research ethics, or publication ethics. If requesting major revision, please articulate the new experiments that are needed.

Reviewer #1: The manuscript presents a timely and relevant investigation into soil-transmitted helminths and schistosomiasis among schoolchildren. The study design, data presentation, and discussion are commendable. However, the entire work would benefit from substantial English language and structural improvement. Additionally, some sections contain redundancy and inconsistent formatting (spacing and punctuation). The authors should also clearly distinguish between STHs and schistosomiasis, as they are biologically and epidemiologically distinct infections. The manuscript would make a valuable contribution to parasitology research.

Response: Thank you!

Reviewer #2: The study presents interesting results and corroborates the need for special attention to the health of schoolchildren for the effective elimination of STH. Furthermore, it also presents data on schistosomiasis, which is also a neglected disease waterborne disease whose elimination is between is among the WHO's Sustainable Development Goals. I suggest that the authors present the schistosomiasis data as additional data to the geohelminthiasis data, and not as a total sum.

Below are some key comments. I am attaching the manuscript with additional comments.

Response: Thank you; we have amended in the revised manuscript (page 14, line 246-248) “The overall prevalence of STH and intestinal schistosomiasis was found to be 42.6% (168/394) (95% CI [(37.8-47.6%]. the prevalence of STH 162 (41.1%) was infected, with a 95% CI of (36.3-46.0%). and *Schistosomiasis* with 13 (3.3%) and a 95% CI of (1.9-5.4%).”

Title: The title suggests reflection on soil-transmitted diseases, which is also observed throughout the text. However, the abstract conclusions already indicate that the study also presents schistosomiasis mansoni, a waterborne disease. Perhaps the title could draw attention to this helminthiasis as well, something like "soil-transmitted helminthiasis and schistosomiasis mansoni" or "transmitted by soil and water," or "helminthiasis in schoolchildren, considering soil-transmitted helminthiasis and schistosomiasis."

Response: Thank you; we have amended the title which read as, “soil-transmitted helminthiasis, Intestinal Schistosomiasis and associated factors” in the revised manuscript

Keywords: It is not advisable to repeat keywords that already appear in the title. Prioritize words that will increase the chances of your text being found during reader searches. Some examples that can be used are: Neglected diseases, intestinal parasitosis, schoolchildren's health, STH, Geohelminthiasis, shistosomiasis

Response: Thank you, we have amended in keywords section (page 3, line 52-53) “Neglected tropical diseases, intestinal parasitosis, schoolchildren's health, STH, Geo-helminthiasis, schistosomiasis” in the revised manuscript

Background:
In the background, it is important to clarify the etiological agent that causes each parasitic disease and the mode of infection. Sometimes the authors mention the names of the parasites, in other they refer to the disease by its name, but without having previously stated what causes it (hookworm).

Line 48: “576-740 million with hookworm and 240 million people infected with schistosomiasis (8).” This might be the point where the authors should include schistosomiasis as something in addition to STH:

Response: Thank you; we have amended in the revised manuscript in the background section the following idea summarised in to short sentences - Hookworm (*Ancylostoma duodenale and Necator americanus*), ascariasis (*Ascaris lumbricoides*), and trichuriasis (*Trichuris trichiura*) are transmitted through skin penetration or ingestion of infective stages from contaminated soil, food, or water, followed by larval migration through the body and maturation in the intestine. Intestinal schistosomiasis, caused by Schistosoma mansoni, occurs when cercariae penetrate the skin during contact with contaminated freshwater and mature in the mesenteric veins, where eggs are released into the intestine and excreted in stool. Like: -(page 4, line 69-72) “Soil-transmitted helminthiasis (STH), caused by *Ascaris lumbricoides*, *Trichuris trichiura*, and hookworms (*Ancylostoma duodenale and Necator americanus*), are a major public health concerns in regions with poor sanitation and inadequate access to clean water. In addition to STHs intestinal schistosomiasis, caused by *schistosoma mansoni* is also prevalent in developing regions.” in the revised Manuscript.

- Furthermore, schistosomiasis, a waterborne parasitic disease, also presents data that should be considered, with approximately 240 million people infected worldwide.

Response: Thank you; we have amended and reconsidered in the revised manuscript

Lines 217 – 224: The authors present high prevalence values when compared to data from North-West Ethiopia, Birbir town, Southern Ethiopia, and Rwanda. However, it is important to clarify whether these studies include schistosomiasis in the STH data or if this inclusion in the present study may be inflating these numbers.

Response: We are grateful for your comments.” In North-West Ethiopia, a study conducted in Birbir town reported that 27.1% of participants tested positive for intestinal parasites, with helminths and protozoa showing prevalences of 21.1% and 7.1%, respectively. In comparison, our current study found an STH prevalence of 41.1% which is higher than the 21.1% helminth prevalence reported in Birbir town. Therefore, our result is not inflated.

Reviewer #3: The manuscript presents a well-executed cross-sectional study assessing the prevalence and intensity of soil-transmitted helminthiasis and associated risk factors among schoolchildren in Hawella Tula Rural district, Ethiopia. The topic is of high public health relevance, particularly in sub-Saharan Africa where STH remains endemic. The authors employ standard diagnostic techniques (Kato-Katz), appropriate statistical analyses (bivariate and multivariable logistic regression), and clearly identify modifiable behavioral and environmental risk factors such as poor hygiene, lack of deworming, and exposure to contaminated water. The sample size is adequate and ethical procedures documented. These strengths make the study a valuable contribution to the literature on NTDs and support its potential to inform targeted interventions.

Response: Thank you for your comments!

However, the manuscript requires revisions before acceptance. Key issues include the absence of a clearly stated hypothesis, missing denominators in prevalence figures, e.g for S. mansoni prevalence estimation, inconsistent references, and formatting gabs in tables and figures. The discussion section would benefit from clearer structure and deeper integration of findings with global STH control strategies, including WHO’s threshold for elimination (<2% moderate-to-heavy intensity infections). Additionally, the conclusion should offer more specific recommendations for public health action such as frequency of MDA required. Provided these revisions are addressed, the manuscript would meet the standards for publication in PLOS Neglected Tropical Diseases. Therefore, I recommend major revision prior to acceptance.

Response: Thank you; we have amended all comments and suggestions in the revised manuscript.

PLOS authors have the option to publish the peer review history of their article ([what does this mean?](https://track.editorialmanager.com/CL0/https:%2F%2Fjournals.plos.org%2Fplosntds%2Fs%2Feditorial-and-peer-review-process%23loc-peer-review-history/1/010f019a6f1b0c1a-035832d6-bfb3-439e-9500-d2328721c901-000000/MpBmXVPZSAOatRWso1NtD3RaHMq5Tov_XKE6CqZQMrA=236)). If published, this will include your full peer review and any attached files.


If you choose “no”, your identity will remain anonymous but your review may still be made public.


**Do you want your identity to be public for this peer review?** For information about this choice, including consent withdrawal, please see our [Privacy Policy](https://track.editorialmanager.com/CL0/https:%2F%2Fwww.plos.org%2Fprivacy-policy/1/010f019a6f1b0c1a-035832d6-bfb3-439e-9500-d2328721c901-000000/A_R8FNj_zg9v9Ia6cR7NNkOX9jERVvYq-Kci5wPRUSw=236).

Reviewer #1: **Yes:** Christianah Oki

Reviewer #2: No

Reviewer #3: No

[NOTE: If reviewer comments were submitted as an attachment file, they will be attached to this email and accessible via the submission site. Please log into your account, locate the manuscript record, and check for the action link "View Attachments". If this link does not appear, there are no attachment files.]

**Figure resubmission:**

While revising your submission, we strongly recommend that you use PLOS’s NAAS tool (https://ngplosjournals.pagemajik.ai/artanalysis) to test your figure files. NAAS can convert your figure files to the TIFF file type and meet basic requirements (such as print size, resolution), or provide you with a report on issues that do not meet our requirements and that NAAS cannot fix.

After uploading your figures to PLOS’s NAAS tool - https://ngplosjournals.pagemajik.ai/artanalysis, NAAS will process the files provided and display the results in the "Uploaded Files" section of the page as the processing is complete. If the uploaded figures meet our requirements (or NAAS is able to fix the files to meet our requirements), the figure will be marked as "fixed" above. If NAAS is unable to fix the files, a red "failed" label will appear above. When NAAS has confirmed that the figure files meet our requirements, please download the file via the download option, and include these NAAS processed figure files when submitting your revised manuscript.

**Reproducibility:**

To enhance the reproducibility of your results, we recommend that authors of applicable studies deposit laboratory protocols in protocols.io, where a protocol can be assigned its own identifier (DOI) such that it can be cited independently in the future. Additionally, PLOS ONE offers an option to publish peer-reviewed clinical study protocols. Read more information on sharing protocols at <https://plos.org/protocols?utm_medium=editorial-email&utm_source=authorletters&utm_campaign=protocols>
