# Supplementary material for: Prevalence, infection intensity, and risk factors of soil-transmitted helminthiasis and intestinal schistosomiasis among schoolchildren in Southern Ethiopia
Source: PLoS Negl Trop Dis. 2026 Mar 18;20(3):e0014115. doi: 10.1371/journal.pntd.0014115 (PMC13016474; doi:10.1371/journal.pntd.0014115)
Supplement: Attachment — Submitted filename: PLOS NTD Reviewers Evalauation revision.docx [file pntd.0014115.s007.docx]

**Reviewer’s Evaluations**

**Prevalence and intensity of soil-transmitted helminthiasis, intestinal schistosomiasis and associated factors among school child Hawella Tula Rural district, Hawassa, Sidama Regional State, Ethiopia.**

| Journal: | *PLOS Neglected Tropical Diseases* |
| --- | --- |
| URL: | https://mc.manuscriptcentral.com/oryx |
| Manuscript Number: | PNTD-D-25-01634R1 |
| Keywords: | Prevalence, Intensity of STH, Schistosoma, Factors; School children,  Southern, Ethiopia |

**Reviewer’s Evaluation 1**

**Evaluation Report**

Kindly enter comments per section of the manuscript **(All comments should be detailed to provide adequate information for authors)**

| General comment | The manuscript addresses an important public health issue by assessing the prevalence and risk factors of soil-transmitted helminths (STHs) and *Schistosoma mansoni* among schoolchildren in southern Ethiopia. The study design, data presentation, and discussion are commendable. However, the entire work would benefit from substantial improvements in the English language and structure. Additionally, some sections contain redundancy and inconsistent formatting (spacing and punctuation). Several sentences are run-on or contain subject-verb agreement errors. Some paragraphs, especially in the Results and Discussions, contain repetitive phrasing and abrupt transitions between STHs and schistosomiasis. References and statistical reporting (percentages with 95% CI) should be uniform. The manuscript would make a valuable contribution to parasitology research. |
| --- | --- |
| Abstract | Please revise punctuation and structure for clarity and replace the semicolon after “and” with a full stop. |
| Author summary | The Author Summary provides relevant background and emphasizes key public health concerns; however, the writing should be improved.   - Line 57: “frequent contact with contaminated water.” - Line 60: *“with A. lumbricoides and followed by T. trichiura being most prevalent”* can be rewritten. Please consider: *“with A. lumbricoides being the most prevalent, followed by T. trichiura.”* - Line 61–63: lists risk factors. However, it can be “eating unwashed vegetables, swimming or bathing in contaminated water, and missing deworming treatment.” - Line 62: “further increased the risk of infection.” - Line 64–65: can be merged with Line 66–67 |
| Introduction | - Line 69: *“transm1itted”* should be *“transmitted* - Line 70–71: Please check the grammar - Line 72: There should be a comma or semicolon after *STHs*, and “intestinal schistosomiasis” should start a new clause. *Schistosoma mansoni* not *schistosoma mansoni* - Line 76: schistosomiasis not schistosomiasi*s* - Line 87: *“Schistosomiasis are widely distributed”* is incorrect   Schistosomiasis is widely distributed and will fit appropriately.   - Line 87–88: The phrase *“living in STH-endemic areas”* seems misplaced if the focus is on schistosomiasis. If you are reporting people at risk of STH and schistosomiasis separately, please clarify. - Line 89: “launched a mass deworming program” - Line 89-90: “school-aged children” - Line 94: *“has never fallen below the World Health Organization (WHO) threshold for low prevalence”* is technically correct but could be rephrased for clarity: *“has consistently remained above the WHO threshold for low prevalence.* - Lines 103–106: please reduce repetition   Soil-transmitted helminthiasis (STH), caused by *Ascaris lumbricoides*, *Trichuris trichiura*, and hookworms (*Ancylostoma duodenale* and *Necator americanus*), is a major public health concern in regions with poor sanitation and inadequate access to clean water. In addition to STHs, intestinal schistosomiasis, caused by *Schistosoma mansoni*, is also prevalent in these regions.  Please ensure references follow the sentence-ending punctuation and are consistent. |
| Methodology | - This looks good |
| Results | - Please confirm the results/figure/table for the data presented. |
| Discussion | - The discussion is rich and well-structured. However, it can be revised. - There are minor spacing issues - The species names (*A. lumbricoides*, *T. trichiura*, Hookworm spp., etc.) should be italicized consistently. - Line 293: *“the findings of the present study was higher”* → should be *“were higher”* - Line 307: Avoid ambiguous phrases like *“some of the possible and likely reasons”* Please choose one - Line 308: *“helminthes eggs”* → should be *“helminth eggs”*. |
| Conclusion | - Line 341: strengthened annual mass drug administration (MDA) is recommended - Line 342: semicolon should be a comma. - Species names: *Schistosoma mansoni* should be italicized |
| Bibliography/References | The reference section is extensive. |
| Final Note | This article, at this stage, needs minor corrections before acceptance |

Please rate the following: (1 = Excellent) (2 = Good) (3 = Fair) (4 = poor)

| Originality | 2 |
| --- | --- |
| Contribution To the Field | 2 |
| Technical Quality | 2 |
| Clarity Of Presentation | 2 |
| Depth Of Research | 2 |

**Recommendations**

Kindly mark with an X

| Accept As It Is |  |
| --- | --- |
| Requires Minor Corrections | **X** |
| Requires Moderate Revision |  |
| Requires Major Revision |  |
| Not Suitable for Further Processing (Please be clear and specific) |  |

**Additional Comments**

Please add any additional comments (Including comments/suggestions regarding online supplementary materials) if any
