# Supplementary material for: Prevalence, infection intensity, and risk factors of soil-transmitted helminthiasis and intestinal schistosomiasis among schoolchildren in Southern Ethiopia
Source: PLoS Negl Trop Dis. 2026 Mar 18;20(3):e0014115. doi: 10.1371/journal.pntd.0014115 (PMC13016474; doi:10.1371/journal.pntd.0014115)
Supplement: Attachment — Submitted filename: Response to Reviewer- FB March 1.docx [file pntd.0014115.s008.docx]

**Response to Reviewers**

**Manuscript ID: PNTD-D-25-01634R1**

**Manuscript Title: Prevalence, Infection Intensity, and Risk Factors of Soil-Transmitted Helminthiasis and Intestinal Schistosomiasis among Schoolchildren in Southern Ethiopia**

Dear Editor and all reviewers

We sincerely thank the Editor and all reviewers for their careful evaluation of our manuscript entitled “Prevalence and intensity of soil-transmitted helminthiasis, intestinal schistosomiasis and associated factors among school child Hawella Tula Rural district, Hawassa, Sidama Regional State, Ethiopia.” We are grateful for the constructive and insightful comments, which have helped us improve the clarity, rigor, and impact of the manuscript. Below, we address each comment point by point. All changes have been incorporated into the revised manuscript, and page and line numbers refer to the revised version.

**Reviewers' comments:**

Reviewer's Responses to Questions

**Key Review Criteria Required for Acceptance?**
As you describe the new analyses required for acceptance, please consider the following:

**Methods**
-Are the objectives of the study clearly articulated with a clear testable hypothesis stated?
-Is the study design appropriate to address the stated objectives?
-Is the population clearly described and appropriate for the hypothesis being tested?
-Is the sample size sufficient to ensure adequate power to address the hypothesis being tested?
-Were correct statistical analysis used to support conclusions?
-Are there concerns about ethical or regulatory requirements being met?

Reviewer #1: The objectives to determine prevalence and associated risk factors of STHs and Schistosoma mansoni among schoolchildren are clearly stated. A cross-sectional study is appropriate for estimating prevalence and assessing associations between risk factors. The study focuses on school-aged children in Hawella Tula Rural District, which is the population at risk for STHs and intestinal schistosomiasis. Ethical approval was obtained for the study.

Response: We sincerely thank the reviewer for their careful evaluation of our manuscript!

iv)What criteria were used for school (site) selection?
Response: “convenient sampling techniques used for sub city selection and all three primary schools within the area were included in the study”.
Query: site selection for SCH should be predicated on risk exposure rather than just convenience sampling. Purposeful sampling is accepted on the grounds that factors driving transmission are well defined and characterized in the area where the survey is being conducted.

Response: We thank the reviewer for this thoughtful comment. We have amended in the revised manuscript methods section (page 7, Line 140-142) “The study site was selected based on the burden of soil-transmitted helminth infections and schistosomiasis, and all three primary schools within the area were included in the study.”
Highlight MDA history in the background.

Response: We thank the reviewer for this thoughtful comment. We have now highlighted the history of the mass drug administration (MDA) program in Ethiopia the Introduction section to provide additional background and context.

**Results**
-Does the analysis presented match the analysis plan?
-Are the results clearly and completely presented?
-Are the figures (Tables, Images) of sufficient quality for clarity?

Reviewer #1: The study reports prevalence, 95% confidence intervals, and associations between infection and risk factors. The prevalence data and risk factors are reported, but some sections contain repetitive phrasing.

**Response:** We thank the reviewer for this comment. We have amended in the revised manuscript results section (page 14-15, Line 245-254)

Reviewer #3: No further comments

**Abstract**

**Reviewer #1:** Please revise punctuation and structure for clarity and replace the semicolon after “and” with a full stop.

Response: We thank the reviewer for this helpful suggestion. We have carefully revised the punctuation and sentence structure throughout the entire manuscript to improve clarity and replaced the semicolon after “and” with a full stop wherever applicable.

**Author summary**

**Reviewer #1** The Author Summary provides relevant background and emphasizes key public health concerns; however, the writing should be improved.

- Line 57: “frequent contact with contaminated water.”

Response: We thank the reviewer for this thoughtful comment; we have amended the revised manuscript Author summary section (page 4, Line 70) “frequent contact with contaminated water.”

- Line 60: *“with A. lumbricoides and followed by T. trichiura being most prevalent”* can be rewritten. Please consider: *“with A. lumbricoides being the most prevalent, followed by T. trichiura.”*

**Response:** We thank the reviewer for this comment we have amended in the revised manuscript Author summary section (page 4, Line 73-74). “With *A. lumbricoides* being the most prevalent, followed by *T. trichiura”*

- Line 61–63: lists risk factors. However, it can be “eating unwashed vegetables, swimming or bathing in contaminated water, and missing deworming treatment.”

Response: We thank the reviewer for this thoughtful comment. we have amended in the revised manuscript Author summary section (page 4, Line 74-75) “eating unwashed vegetables, swimming or bathing in contaminated water, and lack of deworming treatment”

- Line 62: “further increased the risk of infection.”

Response: We thank the reviewer for this. We have amended in the revised manuscript the Author summary section comment (page 4, Line 76). “Further increased the risk of infection.”

Line 64–65: can be merged with Line 66–67

**Response:** We thank the reviewer for this thoughtful comment. we have amended in the revised manuscript Author summary section (page 4, Line 76-80)

**Introduction (Background)**

- Line 69: *“transm1itted”* should be *“transmitted*

Response: Thank you, we have amended in the revised manuscript background section (page 4, Line 82) “transmitted.”

- Line 70–71: Please check the grammar

Response: Thank you, we have amended in the revised manuscript background section (page 4, Line 83-84) “is a major public health concern in regions with poor sanitation and inadequate access to clean water.”

- Line 72: There should be a comma or semicolon after *STHs*, and “intestinal schistosomiasis” should start a new clause. *Schistosoma mansoni* not *schistosoma mansoni*

Response: Thank you, we have amended in the revised manuscript background section (page 4, Line 84) “STHs,”

Response: Thank you, we have amended in the revised manuscript background section (page 4, Line 85) *“Schistosoma mansoni”*

- Line 76: schistosomiasis not schistosomiasi*s*

Response: Thank you, we have amended in the revised manuscript background section (page 5, Line 89) “schistosomiasis”

- Line 87: *“Schistosomiasis are widely distributed”* is incorrect

Schistosomiasis is widely distributed and will fit appropriately.

Response: We thank the reviewer for this thoughtful comment. we have amended in the revised manuscript background section (page 5, Line 100) “STHs is widely distributed, with an estimated 96.7 million people living in STH-endemic areas”

- Line 87–88: The phrase *“living in STH-endemic areas”* seems misplaced if the focus is on schistosomiasis. If you are reporting people at risk of STH and schistosomiasis separately, please clarify.

Response: We thank the reviewer for this thoughtful comment. we have amended in the revised manuscript background section (page 5, Line 99-100) “STHs is widely distributed” with an estimated 96.7 million people living in STH-endemic areas;”

- Line 89: “launched a mass deworming program”

Response: We thank the reviewer. we have amended in the revised manuscript background section (page 5, Line 100) “launched a mass deworming program”

- Line 89-90: “school-aged children”

Response: We thank the reviewer. we have amended in the revised manuscript background section (page 5, Line 100-101). “School-aged children”

- Line 94: *“has never fallen below the World Health Organization (WHO) threshold for low prevalence”* is technically correct but could be rephrased for clarity: *“has consistently remained above the WHO threshold for low prevalence.*

Response: We thank the reviewer for this thoughtful comment. we have amended in the revised manuscript background section (page 5, Line 106-107) “infections has consistently remained above the WHO threshold for low prevalence.”

- Lines 103–106: please reduce repetition

Soil-transmitted helminthiasis (STH), caused by *Ascaris lumbricoides*, *Trichuris trichiura*, and hookworms (*Ancylostoma duodenale* and *Necator americanus*), is a major public health concern in regions with poor sanitation and inadequate access to clean water. In addition to STHs, intestinal schistosomiasis, caused by *Schistosoma mansoni*, is also prevalent in these regions.

Please ensure references follow the sentence-ending punctuation and are consistent.

Response: We thank the reviewer for this thoughtful comment.

Reviewer #3: There is still no testable hypothesis in the revised manuscript. Below is the authors’ response to my previous query:
"Response: We thank the reviewer for this insightful comment. We agree that a clearly stated hypothesis strengthens the study's framework. In response, we have now added a few sentences at the end of the introduction section to address the testable hypothesis (page 5, lines 93-106) in the revised manuscript, which reads: “Despite various efforts to reduce the burden of soil transmitted helminth (STH) infections and schistosomiasis in Ethiopia, the prevalence of these infections has never fallen below the World Health Organization (WHO) threshold for low prevalence. The goal of this threshold is to reduce morbidity from STH infections in preschool-aged children (pre-SAC) and school-aged children (SAC) by lowering the prevalence of moderate- or heavy-intensity infections to less than 1% in SAC. This ongoing burden is driven by factors including rapid reinfection posttreatment, entrenched environmental exposure risks, and complex socio-demographic determinants. As a result, continuous
monitoring of the socio-demographic, environmental, and lifestyle characteristics associated with an increased risk of STH infections is essential. Tailoring interventions and control strategies to meet the specific needs of each community is critical. This study provides evidence on the prevalence and distribution of STH infections and intestinal schistosomiasis among schoolchildren in southern Ethiopia. It highlights a significant public health challenge and aims to inform targeted interventions for clinicians, public health officials, and policymakers to reduce the burden of STH infections and improve child health.”

I suggested rewording to:
" Despite various efforts to reduce the burden of soil‑transmitted helminth (STH) infections and schistosomiasis in Ethiopia, the prevalence of these infections has never fallen below the World Health Organization (WHO) threshold for low prevalence. This threshold aims to reduce morbidity among preschool‑aged and school‑aged children by lowering the prevalence of moderate‑ or heavy‑intensity infections to less than 1% in school‑aged children. The persistent burden is driven by factors such as rapid reinfection after treatment, entrenched environmental exposure risks, and complex socio‑demographic determinants. Continuous monitoring of socio‑demographic, environmental, and lifestyle characteristics associated with increased STH risk is therefore essential, and tailoring interventions to the specific needs of each community remains critical.
This study provides evidence on the prevalence and distribution of STH infections and intestinal schistosomiasis among schoolchildren in southern Ethiopia. It highlights a significant public health challenge and aims to inform targeted interventions for clinicians, public health officials, and policymakers to reduce the burden of STH infections and improve child health. We hypothesize that specific socio‑demographic and environmental factors, such as household sanitation, water access, and parental education, are significantly associated with the risk of STH infection among school‑aged children".

Response: We thank the reviewer for this thoughtful comment. We have amended the revised manuscript background section (pages 5-6, Lines 106-121) based on this recommendation: “….*We hypothesize that specific socio-demographic and environmental factors, such as household sanitation, water access, and parental education, are significantly associated with the risk of STH infection among school-aged children.”*

**Discussion**

Reviewer #1

The discussion is rich and well-structured. However, it can be revised.

**Response:** We thank the reviewer for this comment. We have amended in the revised manuscript

There are minor spacing issues

**Response:** We thank the reviewer for this comment. We have amended in the revised manuscript

The species names (*A. lumbricoides*, *T. trichiura*, Hookworm spp., etc.) should be italicized consistently.

**Response:** We thank the reviewer for this comment. We have amended in the revised manuscript

Line 293: *“the findings of the present study was higher”* → should be *“were higher”*

**Response:** We thank the reviewer for this comment. we have amended in the revised manuscript Discussions section (Page18-19, Line 293-294) “Conversely, it exceeds the 27.1% reported in Birbir town, Southern Ethiopia”

Line 307: Avoid ambiguous phrases like *“some of the possible and likely reasons”* Please choose one

**Response:** We thank the reviewer for this comment. we have amended in the revised manuscript Discussions section (Page 19, Line 305) “possible reasons”

Line 308: *“helminthes eggs”* → should be *“helminth eggs”*.

**Response:** We thank the reviewer for this comment. we have amended in the revised manuscript Discussions section (page 19, Line 306) “helminth eggs”

**General comment**

**Discussion**

The manuscript addresses an important public health issue by assessing the prevalence and risk factors of soil-transmitted helminths (STHs) and *Schistosoma mansoni* among schoolchildren in southern Ethiopia. The study design, data presentation, and discussion are commendable. However, the entire work would benefit from substantial improvements in the English language and structure. Additionally, some sections contain redundancy and inconsistent formatting (spacing and punctuation). Several sentences are run-on or contain subject-verb agreement errors. Some paragraphs, especially in the Results and Discussions, contain repetitive phrasing and abrupt transitions between STHs and schistosomiasis. References and statistical reporting (percentages with 95% CI) should be uniform. The manuscript would make a valuable contribution to parasitology research.

**Response:** We thank the reviewer for this comment. we have amended in the revised manuscript Result section (Page14-15, Line245-254)

**Response:** We thank the reviewer for this comment. we have amended in the revised manuscript Discussions section **(Page 18-19, Line 283-300) and (Page 19-20, Line 308-315)**

**Conclusions**
-Are the conclusions supported by the data presented?
-Are the limitations of analysis clearly described?
-Do the authors discuss how these data can be helpful to advance our understanding of the topic under study?
-Is public health relevance addressed?

Reviewer #1: The conclusions about the prevalence of STHs and Schistosoma mansoni, and the associated risk factors (unwashed vegetables, contaminated water, lack of deworming) are directly supported by the study’s results. The recommendations for school-based deworming, hygiene education, and WASH interventions are also reported. The limitations were clearly described. The discussion emphasizes how risk factors contribute to infection and reinfection dynamics. Yes, public health relevance addressed

Response: We sincerely thank the reviewer for their careful evaluation of our manuscript and for recognizing that the conclusions are well supported by the study findings. We appreciate the positive comments regarding the clarity of the reported risk factors, the public health relevance of the recommendations, and the transparent discussion of study limitations. We are grateful for the reviewer’s thoughtful assessment.

Reviewer #1: Line 341: strengthened annual mass drug administration (MDA) is recommended

Response: We thank the reviewer for this comment. we have amended in the revised manuscript conclusions section (Page 21, Line 334) “strengthened annual mass drug administration (MDA) is recommended”

Line 342: semicolon should be a comma.

We thank the reviewer for this comment. we have amended in the revised manuscript conclusions section (Page 21, Line 334) “interventions,”

Species names: *Schistosoma mansoni* should be italicized

We thank the reviewer for this comment. we have amended in the revised manuscript.

Reviewer #3: No further comments

**Editorial and Data Presentation Modifications?**


Use this section for editorial suggestions as well as relatively minor modifications of existing data that would enhance clarity. If the only modifications needed are minor and/or editorial, you may wish to recommend “Minor Revision” or “Accept”.

Reviewer #1: Minor Revision

Reviewer #3: Please re-edit all tables (1-4). They still appear quite distorted. Ensure the figures are numbered in sequence.

Response: We thank the reviewer for this thoughtful comment. we have amended in the revised manuscript Result section all tables (1-4).
